# A Tiered Approach to Human Synapse Proteomics: Optimized LC-MS/MS Analysis of Whole-Tissue Lysate and Synaptosome Preparations from Frozen Post-Mortem Brain Samples

**DOI:** 10.3390/cells15080736

**Published:** 2026-04-21

**Authors:** Femke C. Roig-Kuhn, Remco V. Klaassen, Frank T. W. Koopmans, Tiara S. Z. Koolman, August B. Smit, Sabine Spijker

**Affiliations:** Department of Molecular and Cellular Neurobiology, Center for Neurogenomics and Cognitive Research, Amsterdam Neuroscience, Vrije Universiteit Amsterdam, De Boelelaan 1100, 1081 HV Amsterdam, The Netherlands; f.c.roigkuhn@vu.nl (F.C.R.-K.); r.v.klaassen@vu.nl (R.V.K.); frank.koopmans@vu.nl (F.T.W.K.); t.s.z.koolman@vu.nl (T.S.Z.K.); guus.smit@vu.nl (A.B.S.)

**Keywords:** shotgun neuroproteomics, frozen tissue, human, synapse proteomics, sTRAP

## Abstract

**Highlights:**

**What are the main findings?**

- Protocol Optimization (sTRAP): The team successfully refined a “one-tube” digestion protocol using 10 µm thin tissue sections and high-temperature (95 °C) solubilization, which ensured consistent protein yields across both healthy and Alzheimer’s disease samples.
- Proteomic Resolution: While whole-tissue lysates are efficient for broad proteomic coverage (>6600 proteins), synaptosome isolation (>6000 proteins) specifically showed 628 enriched proteins—primarily ion channels and transporters—and detected unique synaptic markers (like SHISA6 and GABRG3) that were nearly invisible in the total lysate.

**What are the implications of the main findings?**

- Enhanced Discovery of Low-Abundant Targets: By demonstrating that certain functional proteins (kinases, phosphatases, and receptors) are only confidently quantifiable in synaptosomes, the study provides a roadmap for finding “hidden” drug targets that whole-tissue studies might miss.
- Standardization for Clinical Cohorts: The high reproducibility and low technical variation in the workflow make it a robust candidate for large-scale clinical studies where sample handling must be minimized to maintain data integrity.

**Abstract:**

Recent advancements in neuroproteomics have enabled detailed analysis of protein expression in the human brain, yet resolving synaptic dysfunction—a central feature of many neurological and psychiatric disorders—requires careful methodological consideration. Leveraging the high sensitivity of modern liquid chromatography-tandem mass spectrometry (LC-MS/MS), we evaluated the utility of whole-tissue lysates versus enriched synaptosome preparations for detecting synaptic protein signatures. First, we optimized and standardized a sample preparation protocol for frozen human gray matter (GM) by refining the suspension trapping (sTRAP) digestion method using thin human tissue sections. We accomplished low technical variation by minimizing sample handling and achieved a highly reproducible sample preparation workflow by rigorously applying standardization and randomization across dissection, processing, and LC-MS/MS runs. Second, comparative LC-MS/MS analysis showed that while whole-tissue lysates provide a high-throughput survey of the synaptic proteome, synaptosome isolation is required to investigate synapse-specific proteins to detect alterations at the terminal that are obscured in the soma. Because these methods offer distinct but synergistic levels of information, we recommend a tiered neuroproteomics strategy. This approach utilizes whole-tissue lysates for broad disease-associated screening and consistent quantification in large cohorts, followed by targeted synaptosome proteomics to provide a unique window of insight into synaptic composition and stability. This integrated workflow respects the biological necessity of spatial resolution while maintaining the reproducibility required for robust human brain proteomics. Furthermore, initial tissue-level analysis provides the necessary context to correctly interpret synaptosome data in cases of global synapse loss or gain.

## 1. Introduction

Molecular techniques to study neurodegenerative and neuropsychiatric disorders are becoming increasingly precise and comprehensive. With the advent of high-throughput omics analyses like transcriptomics and proteomics, it is now possible to characterize the molecular signatures that distinguish specific disorders from healthy controls in large patient cohorts. This aids tremendously in uncovering underlying disease mechanisms, as a prelude to researching new treatment options. Yet, quantitative analyses on large cohorts pose many questions on how to best prepare and analyze the samples in a manner tailored to the specific research project.

One of the most direct methods to investigate differences between health and disease in humans is using frozen post-mortem brain tissue, made available by brain banks worldwide, with the goal of increasing the understanding of the human brain [1]. In addition to limitations in the quantity of brain tissue available from brain banks, omics studies face several key challenges. Chief among these are issues related to tissue quality—especially the post-mortem interval—which can lead to degradation of RNA and proteins. Sample heterogeneity, including differences in age, sex, cause of death, medication use, and comorbid conditions, further complicates data interpretation. Technical challenges in sample preparation, such as variability and batch effects, also pose significant obstacles. These factors are especially critical in research on neuropsychiatric disorders, which often lack clear neuropathological markers. Moreover, proteomic alterations in these conditions are typically subtle and highly variable, reflecting the inherent biological complexity of human brain tissue. Careful experimental design, rigorous methodological controls, and minimizing technical variation—particularly during protein extraction and mass spectrometry [2]—are therefore essential to obtain meaningful insights.

Proteomics protocols are widely established in rodent research [3,4]. However, mouse tissue differs from human tissue in several important anatomical and biochemical aspects that should be considered when translating these mouse protocols to human research. Compared to mice, humans exhibit slightly higher neuronal density, increased cortical thickness, and larger neurons [5], along with a substantially greater brain volume driven by a more expanded and folded cerebral cortex. Additionally, human brain tissue contains a higher myelin content than mouse brain tissue [6]. Because myelin is also present in human gray matter [7], the altered protein–lipid composition of human brain tissue necessitates protocol modifications, such as sonication [8], to ensure efficient protein extraction. However, introducing additional steps could increase technical variability between batches. To mitigate this, we sought to adapt existing protocols to improve sample solubilization and preparation for mass spectrometry, while minimizing additional steps that would compromise reproducibility.

In addition to whole-tissue lysate proteomics, measurements on subcellular fractions—coined under the umbrella of spatial proteomics [9]—provides valuable insights into neuronal function and pathology. Because synaptic connections underpin neural network physiology, their dysfunction is a hallmark of various brain disorders. Indeed, synaptic alterations have been associated with neurodegenerative disorders [10,11] and neuropsychiatric disorders [12,13,14]. While whole-tissue lysates offer a broad survey of the proteome, synaptic fractionation is a powerful approach to study disease-specific subcellular regulation. Isolating the synaptic compartment allows the detection of subtle changes in proteins specific to the synapse that would otherwise not be detected in the lysate due to dilution in the whole-tissue context. Historically, synaptic fractionation reduces sample complexity, making it possible to detect more low-abundant synaptic proteins with mass spectrometry. Over the past 6 years, several human proteomics studies focusing on the synapse have been published using different biochemical fractionation approaches (Table S1, [15,16,17,18]), with several studies detecting 5000–9000 proteins. These studies make it important to evaluate the extent of enrichment of synaptic proteins in synaptic isolations relative to whole-tissue lysate preparations. Previous research in mice has revealed brain region-specific differences in synaptic protein enrichment [19]. The extent to which these differences translate to human brain tissue is unclear, as currently there are no studies that compare the whole-tissue lysate proteome to that of the synaptosome with a detection yield of >5000 proteins.

To evaluate the sensitivity of liquid chromatography-tandem mass spectrometry (LC-MS/MS) for detecting synaptic proteins in human whole-tissue lysates versus synaptosome preparations, we first implemented a standardized and randomized workflow designed to minimize sample handling and reduce batch effects. Limiting sample handling enhances reproducibility and increases the feasibility of large-scale studies with several hundreds of samples [20,21,22]. Here, we adapted a revised version of the original ProtiFi suspension-trapping (S-TRAP) mini spin column digestion protocol 4.1 previously described by our lab [3]. This revised ‘sTRAP’ version replaced the commercial ProtiFi S-TRAP column with a low-cost plasmid DNA micro-spin column, which demonstrated superior performance in number of identified proteins and peptides, as well as lower coefficients of variation (CoV) in mouse tissue. With the aim of optimizing reproducible proteomic profiling of human brain tissue, we adapted the existing mouse sTRAP workflow to process micrometer-thin tissue sections in a streamlined one-tube reaction. We systematically characterized the influence of incubation conditions and centrifugation on extract composition and explored the impact of variable tissue input levels on tryptic digestion efficiency and the depth and consistency of protein-level quantification. Throughout the workflow, the sequence of dissection, sample preparation, and LC-MS/MS analysis were carefully arranged and randomized to avoid potential confounding factors. Finally, we assessed the added benefit of isolating the synaptic fraction for enhancing the detection of low-abundance synaptic proteins in a cohort of control donors.

Together, we describe (1) an optimized digestion protocol to maximize peptide and protein yield for LC-MS/MS using thin human post-mortem cryo-sections; and (2) a comparison between whole-tissue lysate and synaptosome proteomics, exploring the added advantages of enriching for synapses given recent improvements in mass spectrometry detection sensitivity. By outlining the rationale behind each methodological step, we provide a practical guide for researchers to design and execute robust and reproducible quantitative proteomic analyses on post-mortem human brain tissue

## 2. Materials and Methods

### 2.1. Tissue Collection from Human Brain Samples

Tissue was obtained from the Netherlands Brain Bank (NBB). Informed consent was obtained from all donors and their next of kin for the use of material and clinical data for research purposes, as outlined in https://www.brainbank.nl/about-us/ethics/ (accessed on 14 April 2026). The NBB autopsy procedures and the use of tissue for research were approved by the Ethics Committee of Amsterdam UMC (registered with the US Office of Human Resource Protections as IRB00002991 under Federal wide Assurance number 00003703), location VUmc, Amsterdam, the Netherlands, albeit that, according to the Dutch Act on Medical Research Involving Human Subjects (WMO), no ethical approval is required for the collection or use of donated brain tissue.

For optimizing the proteomics workflow, we used tissue from the most anterior part of the superior frontal gyrus (SFG)—comprising brain sections 2–7, as presented in the Adult Human Allen Brain Reference Atlas [23], overlapping with the medial and lateral subdivision of Brodmann areas 9 and 10—of two replicate tissue samples from a non-demented control donor (NDC), and two replicate tissue samples from a donor diagnosed with (presenile) Alzheimer’s disease (AD) and (putative) comorbid schizophrenia (Supplemental File S1, clinical information sheet). For analysis of whole-tissue lysates vs. synaptosome preparations, we used the most anterior part of the SFG from 8 NDC donors (Table 1). For all samples, gray matter (GM) was collected from tissue sections (2–10 mg cut at 10 µm for whole-tissue lysate; ~25 mg cut at 50 µm for synaptosome preparations) using a cryostat.

### 2.2. Subcellular Fraction Isolation

Synaptosomes were isolated following the procedures described by Pandya et al. [19] and Li et al. [24], adapted from the original protocol [25] with minor modifications. In short, cortical GM tissue (~25 mg, sectioned at 50 µm) was homogenized in 2 mL ice-cold homogenization buffer (0.32 M sucrose, 5 mM HEPES pH 7.4) using a Teflon/glass homogenizer (Schuett homgenplus; Schuett-biotec 9651560, Göttingen, Germany) set at 900 rpm for 12 strokes. For a detailed step-by-step description, see Supplemental Protocol S2. It should be noted that multiple considerations could be taken along for every step during synaptosome isolation, as seen in ref. [26]. The resulting homogenate was collected and centrifuged at 1000× *g* for 10 min (4 °C) to spin down the nuclear membranes. The supernatant (S1) was placed on a discontinuous sucrose gradient consisting of 0.85 and 1.2 M sucrose. After ultracentrifugation at 187,000× *g* at 4 °C for 2 h, the interphase disk between the sucrose concentrations—containing the synaptosome fraction—was collected. After centrifugation (18,000× *g* (Eppendorf 5810, Hamburg, Germany) at 4 °C for 30 min), the pellet was resuspended in homogenization buffer, and the samples were set aside for sTRAP.

### 2.3. MS Sample Preparation Using Suspension Trapping (sTRAP)

MS sample preparation was performed following the DNA micro spin column suspension trapping (sTRAP) protocol as described by our lab previously [3], with several adaptations to accommodate for human tissue samples.

Human brain sections (~10 mg for the optimization of the whole-tissue lysate extraction, and 2–5 mg for comparison to the synaptosome fraction) were extracted in 500 µL sTRAP lysis buffer (5% SDS, 50 mM Tris-HCl (pH 8.0), 5 mM tris(2-carboxyethyl)phosphine (TCEP), and 20 mM 2-chloroacetamide (2-CAA)) by incubation in a ThermoTop-covered Thermomixer set to 1700 rpm, either at 55 °C for 30 min or at 95 °C for 15 min, followed by 55 °C for 15 min. This one-pot extraction procedure not only homogenized the thin tissue sections but also immediately reduced disulfide bonds and alkylated the free sulfhydryl residues for downstream MS analysis. Insoluble debris was cleared from the tissue lysates by centrifugation at 20,000× *g* for 10 min at room temperature (RT). For lysates belonging to the whole-tissue optimization experiment, aliquots were collected both before and after centrifugation, providing the ‘pre-centrifugation’ and ‘post-centrifugation’ fractions, respectively. Synaptosome samples were processed using the same procedure, with a final extraction volume of 70 µL and a similar extraction condition of 95 °C for 15 min, followed by 55 °C for 15 min. The protein concentration of lysates was determined using the bicinchoninic acid (BCA) protein assay.

For the sTRAP workflow, either 50 µg (extraction optimization: four replicates for each sample) or 10 µg (synaptosome proteomics: for each donor) of total protein was used as an input (in 50 µL sTRAP lysis buffer). For the trypsin/Lys-C digestion efficiency test, pooled whole-tissue lysates were serially diluted to provide an sTRAP protein input of 50 µg, 25 µg, 12.5 µg, and 5 µg. Samples were acidified to a final concentration of 1.1% phosphoric acid (12% stock solution), mixed with six volumes of binding/washing buffer (90% methanol in 100 mM Tris-HCl pH 8.0), and loaded onto a plasmid DNA micro column (HiPure from Magen Biotechnology—C13011, Guangzhou, China, see ref. [3]). The protein particulate was retained on the column upon centrifugation at 1400× *g* for 1 min and the columns were washed four times with binding/washing buffer. Columns were transferred to new LoBind tubes (Eppendorf, Hamburg, Germany), supplemented with either 1 µg trypsin/Lys-C (extraction optimization; Promega—V507A) or 0.4 µg trypsin/Lys-C (synaptosome proteomics) in 50 mM NH_4_HCO_3_, and incubated overnight at 37 °C in a humidified incubator. Tryptic peptides were eluted and pooled by subsequent addition of 50 mM NH_4_HCO_3_, 0.1% formic acid, and 0.1% formic acid in acetonitrile. The collected peptides were dried by SpeedVac and stored at −80 °C.

For a detailed step-by-step description of the optimized protocol, see Supplemental Protocol S1.

### 2.4. LC-MS Analysis

Each sample of tryptic digest was redissolved in 0.1% formic acid, and the peptide concentration was determined by tryptophan fluorescence assay [27]. Peptides were loaded onto an Evotip Pure (Evosep, Odense, Danmark) according to the manufacturer’s instructions, as 75 ng for the extraction optimization samples and 150 ng for the synaptosome proteomics samples. Peptides were separated by a standardized 30 samples per day method on the Evosep One liquid chromatography system, using a 15 cm × 150 μm reverse-phase column packed with 1.5 µm C_18_-beads (EV1137 from Evosep) connected to a 20 µm ID ZDV emitter (Bruker Daltonics, Leiderdorp, The Netherlands).

Peptides were electro-sprayed into the timsTOF Pro 2 mass spectrometer (extraction optimization; Bruker Daltonics) or the timsTOF HT mass spectrometer (whole-tissue lysate vs. synaptosome proteomics; Bruker Daltonics, Leiderdorp, The Netherlands) equipped with CaptiveSpray source and measured with the following settings: scan range 100–1700 m/z; ion mobility 0.65 to 1.5 Vs/cm^2^; ramp time 100 ms; accumulation time 100 ms; and collision energy decreasing linearly with inverse ion mobility from 59 eV at 1.6 Vs/cm^2^ to 20 eV at 0.6 Vs/cm^2^. Operating in dia-PASEF mode, each cycle took 1.38 s and consisted of 1 MS1 full scan and 12 dia-PASEF scans. Each dia-PASEF scan contained two isolation windows, which in total covered 300–1200 m/z and ion mobility 0.65 to 1.50 Vs/cm^2^. Dia-PASEF window placement was optimized using the py-diAID tool [28]. Ion mobility was automatically calibrated at the start of each sample (calibrant m/z, 1/K0: 622.029, 0.992 Vs/cm^2^; 922.010, 1.199 Vs/cm^2^; 1221.991, 1.393 Vs/cm^2^). Samples were processed in a randomized manner to prevent batch effects by MS run order (Supplemental Figure S1).

### 2.5. LC-MS Data Analysis

The dia-PASEF raw data were processed with DIA-NN (version 1.8.1; [29]). An in silico spectral library was generated from the Uniprot human reference proteome (Swiss-Prot and TrEMBL, canonical and additional isoforms, release 2023–02 (extraction optimization) and 2024–02 (whole-tissue lysate vs. synaptosome proteomics)) using trypsin/P digestion and at most 1 missed cleavage. Fixed modification was set to carbamidomethylation (C), and variable modifications were oxidation (M) and N-terminal M excision (at most 1 per peptide). The peptide length was set to 7–30, precursor charge range was set to 2–4, and precursor *m*/*z* range was limited to 280–1220. Both MS1 and MS2 mass accuracy were set to 15 ppm, the scan window was fixed at 9, and the precursor False Discovery Rate (FDR) was set to 1%. Heuristic protein inference was disabled, protein identifiers (isoforms) were used for protein inference, and double-pass mode was enabled. RT-dependent cross-run normalization was enabled with the robust LC quantification strategy. Match-between-runs (MBR) was enabled, while all other settings were left as default.

MS-DAP (version 1.2.2; [30]) was used for downstream analysis. Peptide-level filtering was configured to retain only peptides that were confidently identified in at least 75% of the samples per group. Peptide abundance values were normalized using the VSN algorithm, followed by protein-level mode-between normalization. This approach was shown to be robust across a wide range of datasets, including those with strongly asymmetric protein abundance differences between experimental conditions [30]. Differential expression analysis was performed in a between-subject manner by the DEqMS algorithm [31]. The log_2_ foldchange (FC) threshold was estimated by bootstrap analysis for the optimization part (Supplemental File S1, “Bootstrapping”), as well as for the quantitative comparison of synaptosome vs. lysate (−0.208 > log_2_ FC > 0.208) and resulting *p*-values were adjusted for multiple testing using the Benjamini–Hochberg FDR procedure (FDR cutoff of 1%). The effect size of a regulated protein represents the ratio of relative (log_2_) foldchange to the mean abundance variance across replicates. Differential expression analysis for synaptosome vs. lysate samples was performed accounting for relevant covariates (sex, age; see Table 1). Gene set enrichment analysis was done using the online tools ShinyGO (v0.85.1 [32]) and SynGO (v1.3 [33]), always using the complete list of confidently identified proteins as the background. For SynGO, we focused on gene ontology terms for cellular components in the synapse. ShinyGO was used with standard settings (FDR cutoff 5%) except for increasing the minimal pathway size from 2 to 3 proteins. Venn diagrams were made with InteractiVenn [34].

## 3. Results

**Figure 1:**
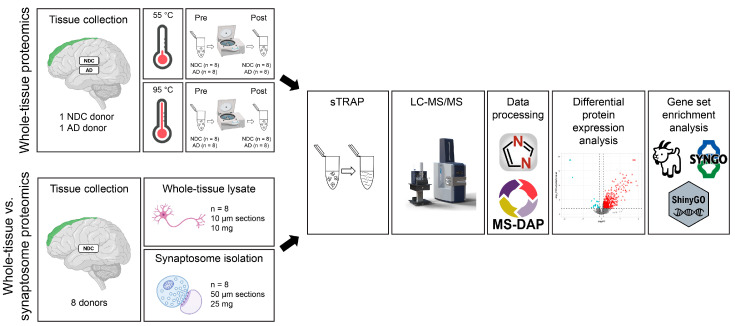
Overview of the proteomics analyses. For proteomics of the extraction optimization (above; “Whole-tissue proteomics”), two samples were obtained from each donor (10 µm sections, 10 mg per sample). Tissue extraction was performed at 55 °C and 95 °C (*n* = 4 per condition; 2 NDC and 2 AD serial tissue samples). Protein samples were collected before and after centrifugation, yielding *n* = 8 replicates per condition. Samples were then prepared for mass spectrometry with sTRAP, and data were processed and analyzed. For the subcellular fraction isolation (below; “Whole-tissue vs. synaptosome proteomics”), two samples were collected per NDC donor: one for whole-tissue lysate proteomics (*n* = 8, 10 µm sections, 10 mg per sample) and one for synaptosome proteomics (*n* = 8, 50 µm sections, 25 mg per sample). Samples were then prepared for mass spectrometry with sTRAP, and data were processed and analyzed. NDC = non-demented control donor; AD = donor with Alzheimer disease and comorbid schizophrenia; Pre = pre-centrifugation; Post = post-centrifugation; sTRAP = suspension trapping; LC-MS/MS = liquid chromatography-tandem mass spectrometry.

In this study, we employed tissue sectioning to selectively collect GM from frozen human brain samples. This method is relatively straightforward and allows for precise anatomical targeting. Moreover, the use of thin (10 µm) tissue sections results in a high surface area-to-volume ratio, which facilitates efficient chemical solubilization. We first optimized our pre-MS workflow on thin GM sections using a selected set of donors (*n* = 4) and subsequently applied this protocol to compare whole-tissue lysates with synaptosome-enriched preparations of NDC donors (*n* = 8) (Figure 1).

### 3.1. Optimization of the sTRAP Micro Spin Column Digestion Protocol for LC-MS/MS Proteomics Analysis of Sectioned Post-Mortem Tissue

To perform neuroproteomic analyses of fresh-frozen human GM cortical tissue using a streamlined one-tube workflow with minimal sample handling, we adapted the recently published sTRAP micro spin column digestion protocol [3] to our sample input type (human GM) and quantity. We first optimized the lysis temperature and SDS buffer volume, changed the alkylation reagent [35], and tested trypsin/Lys-C digestion conditions to ensure optimal protein solubilization and digestion. Protein solubilization is critical for ensuring replicability of samples, as variation in input material largely impacts experimental outcomes. This is specifically important in neurodegenerative research, where protein aggregates (e.g., plagues, tangle) are frequently observed and often constitute key pathological features for molecular analysis [36]. To account for these factors in the optimization part of our study, we used protein samples derived from 10 mg tissue to avoid exceeding solubilization limits. Moreover, tissue was collected from an NDC and AD donor, anticipating that AD samples might present a higher degree of protein aggregation and potentially affect solubilization efficiency.

We first tested qualitative aspects of the solubilization protocol, focusing on SDS buffer volume and solubilization temperature. While mechanical disruption methods like sonication are highly effective for tissue lysis, we prioritized a chemical-only approach to maximize protocol accessibility and minimize the need for specialized instrumentation across different laboratory settings. Therefore, we aimed at improving the chemical solubilization in SDS buffer by making the collected tissue sections thinner (10 µm), thereby increasing the surface area in contact with the buffer. Furthermore, we used a relatively high SDS buffer volume (500 µL), as this was sufficient to solubilize all the tissue without reaching the 1.5 mL Eppendorf tube volume limit. We experienced that lower volumes resulted in incomplete dissolution into the SDS buffer. The optimal temperature for solubilization was 95 °C for all sample types (NDC and AD). However, this caused an undesired pressure build-up during heating. To mitigate this, the temperature was set to 95 °C for 15 min, followed by 55 °C for another 15 min, maintaining the original 30-min solubilization time.

Next, we quantified protein yield from a 10 mg tissue sample to determine the maximum amount of protein for digestion and calculated the amount of trypsin/Lys-C accordingly to represent the lowest possible enzyme–protein ratio of 1:50. A 10 mg tissue sample represented the maximum tissue weight acquired during tissue collection, with average samples ranging between 2 and 5 mg. BCA assays showed no substantial difference in protein content between NDC and AD samples (Supplemental Figure S2), with approximately 9% of the tissue weight corresponding to protein (± 0.9 mg protein per 10 mg GM tissue). Based on this, a fixed quantity of 1 µg trypsin/Lys-C was applied to all samples, maintaining an enzyme-to-protein ratio within the commonly used range of 1:10 to 1:25 (w/w) [3].

Subsequently, we tested the effectiveness of the 95 °C solubilization step during protein extraction compared to 55 °C in terms of protein yield, as measured quantitatively by MS analysis. Furthermore, we evaluated whether centrifugation after extraction was beneficial to obtain a more representative sample from a larger protein pool, as this step could remove larger insoluble aggregates and make the sample more homogeneous. On the other hand, it could result in the loss of specific proteins. For these tests, we compared technical replicates of the previous NDC and AD donors that were either centrifuged or not after solubilization at 55 °C or 95 °C (two temperature settings, each with *n* = 2 tissue replicates, each with *n* = 4 sample replicates (pre-centrifugation, post-centrifugation), yielding eight technical replicates per donor per condition).

**Figure 2:**
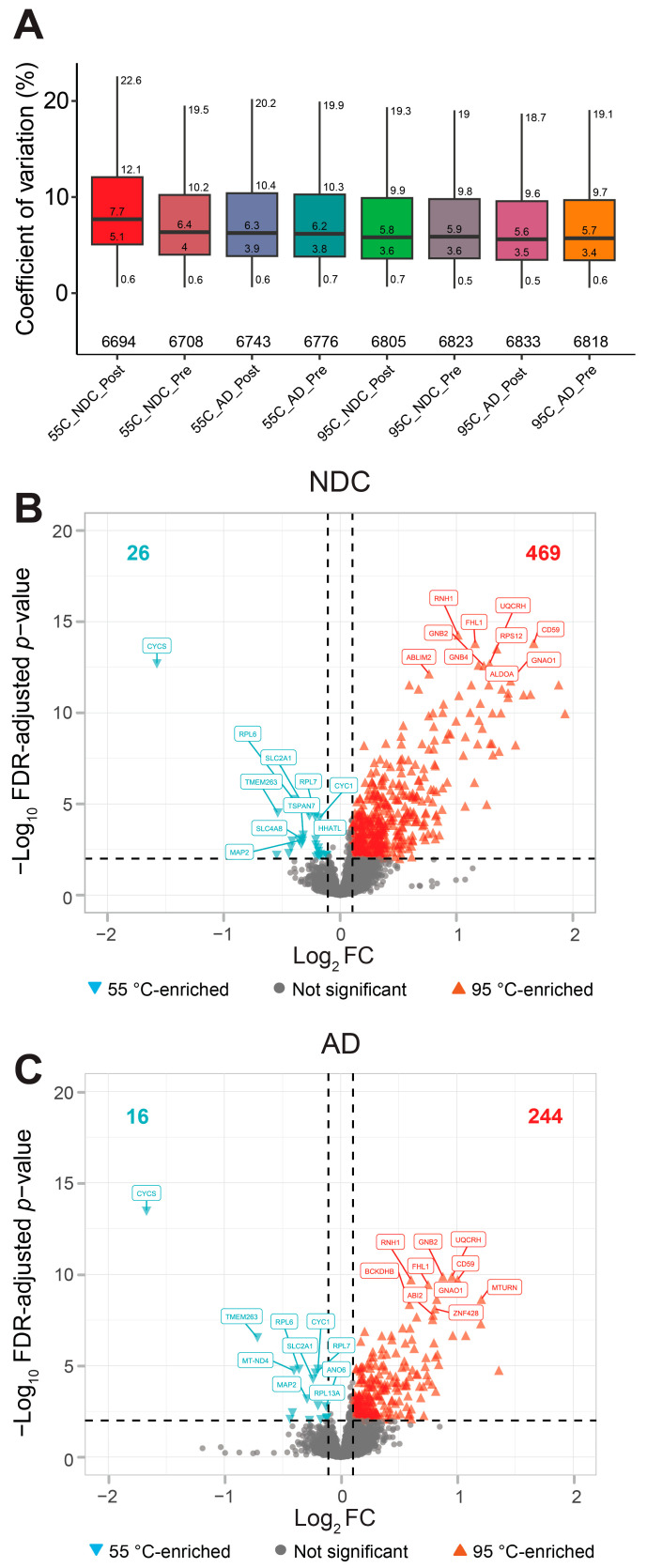
Quality control parameters for one-tube tissue lysate protocol. (**A**) Protein-level coefficient of variation plots of all sample conditions. Values of quartiles and whiskers, as well as the total number of proteins are indicated. A higher temperature during solubilization (95 °C) resulted in a small reduction in coefficient of variation (CoV). (**B**,**C**) Volcano plot of DEqMS results showing the comparison of protein levels after solubilization at 55 °C vs. 95 °C prior to centrifugation for NDC (**B**) and AD (**C**) samples. In total, two tissue replicates (**A**,**B**) and four sample replicates (1–4) were taken per condition, resulting in eight technical replicates per donor. Gene symbols are shown for the top 10 proteins with lowest FDR-adjusted *p*-value (<0.01; horizontal dashed line) for both 55 °C-enriched and 95 °C-enriched. The total number of differentially expressed proteins (vertical dashed line; >|±0.10|) is indicated at the top (left, turquoise; right, red). Pre = pre-centrifugation; Post = post-centrifugation; NDC = non-demented control donor; AD = donor with Alzheimer disease and comorbid schizophrenia. For a complete list of proteins, see Supplemental File S1.

Solubilization at 95 °C yielded approximately the same protein and peptide counts compared to 55 °C in NDC and AD, along with more consistent results in yield (Supplemental Figure S3). Added benefits of solubilization at 95 °C were the increased detection of peptides with modified cysteine residues (11.0–11.9% at 55 °C to 15.9% at 95 °C; Table S2), indicating improved reduction–alkylation efficiency, and the slight decrease in coefficient of variation, apparently reducing (technical) variability between samples (Figure 2A). Higher temperature solubilization led to increased abundance levels (enriched at 95 °C) for a substantial portion of the detected proteins (NDC: 469/5741 = 8.64%; AD: 244/5701 = 4.28%), while only a small fraction showed decreased abundance levels (enriched at 55 °C; NDC: 26/5741 = 0.45%; AD: 16/5701 = 0.28%) (Figure 2B,C). To determine whether the shifts observed in the volcano plots reflected a genuine increase in proteomic recovery rather than a compositional artifact of equal peptide loading, we compared the number of unique peptides identified per protein at both temperatures (Supplemental Figure S4). This analysis, visualized via MA and density plots, confirms that the 95 °C solubilization step significantly broadens the detectable peptide pool across the dynamic range of the proteome. In addition, the proteins for which fewer than two peptides were detected and/or in less than 75% of the samples per group, i.e., those considered not detected at 55 °C (NDC: 168/168 + 5741 = 2.8%; AD: 132/132 + 5701 = 2.26%) or 95 °C (NDC: 79/79 + 5741 = 1.4%; AD: 100/100 + 5701 = 1.72%), were low, indicating that solubilization in this one-tube reaction was optimal and independent of sample type, most likely due to the very thin tissue sections (10 µm) creating sufficient exposure to the chemical solubilization. To explore whether there are consistent protein sets that were not detected at either solubilization temperature, we selected the proteins in overlap between NDC and AD lost at 55 °C (*n* = 43) and at 95 °C (*n* = 11) for gene set enrichment using the gene ontology tool ShinyGO [32]. No enrichment was found. Solubilization temperature does not favor specific proteins. Together, these findings indicate that the 95 °C solubilization step facilitated a higher recovery of protein species compared to the 55 °C. By enhancing the recovery of difficult-to-extract protein subsets without introducing stochastic variability, this optimized temperature setting contributes to a more representative and reproducible starting material for downstream LC-MS/MS analysis. Hence, we selected 95 °C as the solubilization temperature for the subsequent whole-tissue lysate vs. synaptosome proteomics experiment (see Section 2 and Supplemental Protocol S1).

**Figure 3:**
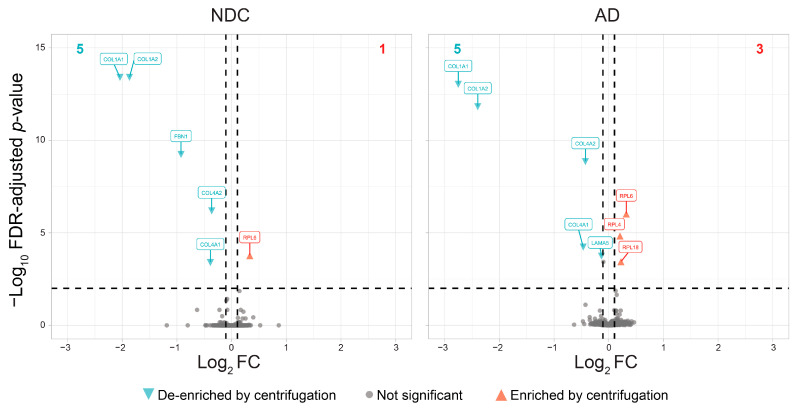
Protein (de-)enrichment by centrifugation of samples. Volcano plots showing the DEqMS contrast of pre- vs. post-centrifugation samples at 95 °C, for control samples (NDC, **left**) and AD samples (AD, **right**). Two tissue replicates and four sample replicates were taken per condition, resulting in eight technical replicates per donor for pre- vs. post-centrifugation. Gene symbols are shown for the significant proteins that are centrifugation-depleted (turquoise) or centrifugation-enriched (red) for FDR-adjusted *p*-values < 0.01 (horizontal dashed line). The total number of differentially expressed proteins (colored; vertical dashed line; >|±0.10|) is indicated at the top (**left**, turquoise; **right**, red). Overall, the difference in protein abundance due to centrifugation is minimal, and greatly similar between the NDC and AD samples. For a complete list of proteins, see Supplemental File S1.

Subsequent centrifugation of the samples did not lead to substantial protein loss, regardless of solubilization temperature or sample type (NDC or AD) (Figure 2). At both 55 °C (Supplemental Figure S5) and 95 °C (Figure 3), the few proteins that were less abundant due to centrifugation included collagens (95 °C: NDC: 5/5800 proteins = 0.09%; AD: 5/5816 proteins = 0.09%), while some ribosomal proteins appeared paradoxically more abundant (95 °C: NDC: 1/5800 proteins = 0.02%; AD: 3/5816 proteins = 0.05%) (Figure 3). The proteins that were not confidently detected, i.e., those considered truly “lost” proteins after centrifugation, represented a small fraction of the total protein pool (95 °C: NDC: 104/104 + 5800 = 1.8%; AD: 92/92 + 5816 = 1.6%; Supplemental File S1). Similarly, the proportion of proteins confidently detected only after centrifugation (“gained” by centrifugation) was comparable across samples (95 °C: NDC: 92/92 + 5800 = 1.6%; AD: 98/98+5816 = 1.7%; Supplemental File S1). The proteins in overlap between NDC and AD not detected in the pre-centrifugation (*n* = 12) and post-centrifugation (*n* = 15) samples at 95 °C were very low, suggesting that these differences likely reflect stochastic detection of low-abundant proteins rather than systematic effects of the centrifugation step. Although overall protein loss was negligible, we decided to retain the centrifugation step in the protocol to mitigate the risk of biased sampling from the extracted protein pool by removing potential debris resulting from the tissue collected.

**Figure 4:**
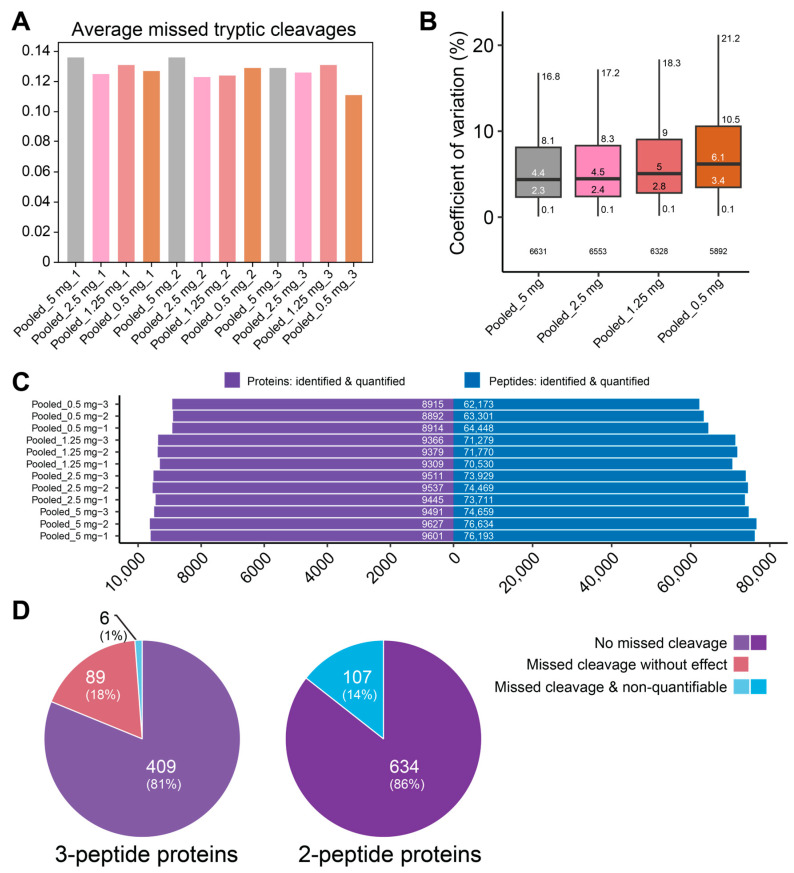
Quality controls for trypsin/Lys-C digestion efficiency. (**A**) Average missed tryptic cleavages of samples simulating 5.0, 2.5, 1.25, and 0.5 mg tissue weight input (50, 25, 12.5, and 5 µg protein equivalent for sTRAP), measured in triplicate (1–2–3) after digestion with 1 µg trypsin/Lys-C (ratio of enzyme-to-protein = 1:5, 1:12.5, 1:25, and 1:50, with 1:20 being the optimal ratio). (**B**) Protein-level coefficient of variation in the previous samples. Values of quartiles and whiskers, as well as the total number of proteins are indicated. (**C**) Protein and peptide counts of all sample conditions. The number of detected peptides and proteins increased with increasing simulated input weight. Note that protein counts here represent the total identification depth (≥1 peptide/protein) to illustrate the full dynamic range of the MS-DAP output across input levels. This differs from the filtered quantification depth (≥2 peptides/protein, detected in 75% of samples) reported in our comparative analyses, which yielded approximately 5700–5800 proteins. (**D**) The number of missed trypsin cleavage affecting the number of quantifiable proteins (becoming unquantifiable, blue; no effect, pink), and proteins not affected (purple) were evaluated when restricting the DIA-NN analysis from one to zero missed cleavages for proteins represented by low peptide numbers (2 and 3).

The final step in optimizing our protocol involved validating the consistency of trypsin/Lys-C digestion when processing samples of variable input levels, reflecting the range of tissue weights that was observed while collecting samples from the gray matter of human tissue blocks. For this, samples from the previous experiments were pooled and subsequently diluted to simulate a series of samples without biological variability, and with a tissue weight equivalent to 5 mg, 2.5 mg, 1.25 mg and 0.5 mg. The sTRAP workflow was performed on an equal fraction of each sample using a fixed amount of trypsin/Lys-C (1 µg). Digestion efficiency, reported by DIA-NN as the “average missed tryptic cleavages” statistic, was found to be consistent for all simulated input weights (Figure 4A), validating the use of a singular trypsin/Lys-C condition for a wide range of protein inputs. The overall number of missed trypsin cleavage sites was low, with 11.7% of all measured peptides and 9.0% of peptides detected in 100% of samples (Table S3). However, when examining the depth of the measured proteome, we observed that the number of identified peptides and proteins gradually decreased—alongside an increase in coefficient of variation—with a reduction in the sTRAP protein input (Figure 4B,C), specifically for protein inputs of 0.5 mg tissue (5 µg protein equivalent). This reduction in peptide and protein IDs should not originate from a reduction in total MS load, as a peptide-level normalization was performed prior to MS measurement. A possible explanation can be found in the relative increase, and therefore load contribution, of peptides that are derived trypsin/Lys-C itself through autolysis at higher enzyme to protein ratios. Alternatively, relative surface losses of specific peptides could become more prominent during the sTRAP workflow at lower input levels, as observed from the increase in variation in these samples. This observed variability of peptide and protein IDs across the simulated tissue levels strongly encourages the use of protein-level normalization prior to digestion (e.g., performing the BCA assay on tissue lysates) to ensure equal loading for sTRAP.

In addition, we evaluated the impact of missed cleavages on protein quantification by assessing the number of proteins that would have been unquantifiable (i.e., represented by fewer than two peptides) if zero missed cleavages were allowed (Figure 4D). This analysis specifically focused on proteins represented by a low number of peptides, as these are most susceptible to loss of quantification. We found that only a relatively small number of proteins—107 (14%) proteins represented by two peptides and six (1%) proteins represented by three peptides—benefited from allowing the standard DIA-NN missed-cleavage setting of one (Figure 4D).

Based on the results of our pilot experiments, we established the following optimized protocol: (1) Using samples of 2–10 mg tissue weight, protein solubilization was performed in 500 µL 5% SDS buffer using 2-CAA for irreversible cysteine alkylation at 95 °C for 15 min, with a 15 min cooldown to 55 °C to avoid safe-lock tubes from opening due to pressure build-up. (2) Extracted proteins were centrifuged to ensure a more representative sample by removing potential aggregates. (3) Trypsin/Lys-C digestion was carried out using a fixed trypsin/Lys-C-to-estimated protein ratio of 1:25 for samples derived from tissue weight of 5–10 mg whenever feasible. For each of the specific steps, see Supplemental Protocol S1. Optimal sampling is achieved by collecting multiple tissue slices to reach a total input of 2–10 mg, thereby ensuring balanced representation of the tissue block; however, this depends on the size and geometry of the available tissue. When only smaller amounts of tissue (e.g., 0.5–1.5 mg) can be collected, we still recommend solubilization in 500 µL, but this would result in iterative loading of the protein suspension on the sTRAP column to reach the minimal input of 12.5 µg protein (1.25 mg tissue-equivalent; cf. Figure 4). The specifics of this have not been tested here.

### 3.2. Subcellular Fraction Isolation: Synaptosome Proteomics

Proteomic technologies have advanced rapidly, particularly in terms of sensitivity and the quantification of proteins relative to the input material required. Subcellular fractionation—such as synaptosome isolation in neuroscience studies [37]—has been commonly employed to reduce sample complexity and enrich relevant protein subsets [38], thereby overcoming limitations in detection and quantification. However, with current mass spectrometers now capable of identifying ~10,000 proteins, such detection limits have become less of an issue. In this context, we evaluated whether isolating synaptosome fractions would still provide an advantage in detecting low-abundant synaptic proteins compared to whole-tissue lysates. To test this, we performed a quantitative proteomics analysis comparing whole-tissue lysates and synaptosome-enriched lysates from the same set of NDC donors within the NBB cohort (*n* = 8; 4 females, see Table 1). We collected 5.0 mg GM (10 µm sections) for whole-tissue lysate analysis and 25.0 mg GM (50 µm sections). Synaptosomes were isolated using an in-house subcellular fractionation protocol (Supplemental Protocol S2). The sTRAP solubilization process was adapted slightly to accommodate the distinct nature of the synaptosome samples but was otherwise followed as previously described (Supplemental Protocol S1). All samples were randomized to avoid batch effects.

**Figure 5:**
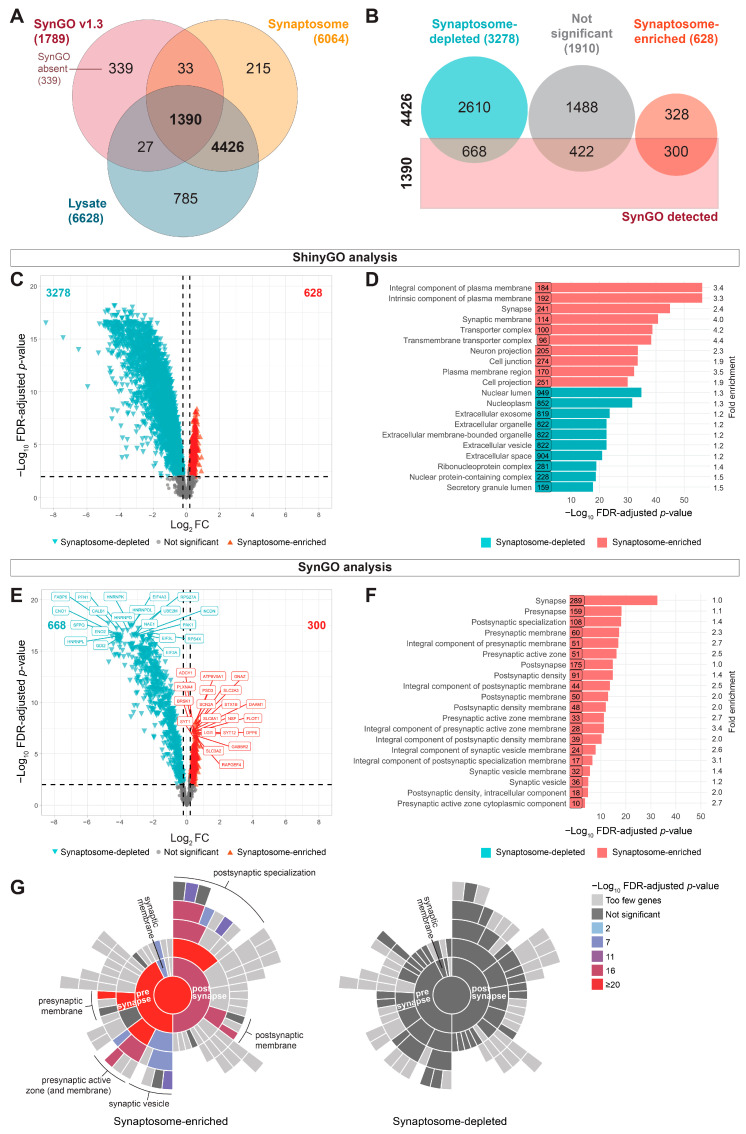
Enrichment of synaptic proteins in the synaptosome fraction. (**A**) Venn diagram showing the quantified protein coverage (defined as ≥ 2 peptides per protein and detected in 75% of samples per group) and the proportion of SynGO-annotated synaptic proteins (“SynGO detected”), as well as SynGO proteins not detected in whole-tissue lysate (“Lysate”) and synaptosome (“Synaptosome”) samples. (**B**) Diagram showing the categorization of the quantified proteome in the contrast of whole-tissue lysate vs. synaptosome samples (synaptosome-depleted, turquoise; synaptosome-enriched, red; not significant, gray; see panel (**C**)), and whether or not the proteins were annotated in SynGO (1390 and 4426 resp. in panel (**A**)). (**C**) Volcano plot showing the contrast between whole-tissue lysate and synaptosome for NDC donors (*n* = 8 for whole-tissue lysate vs. *n* = 8 for synaptosomes). The largest group of proteins shows depletion (turquoise) in the synaptosome samples, while a smaller number of proteins is enriched (red). The total number of differentially expressed proteins is indicated at the top (**left**, turquoise; **right**, red). The top 20 significant proteins on each side are labeled (FDR-adjusted *p*-values < 0.01; horizontal dashed line). (**D**) Bar graph showing the top 10 overrepresented GO annotations for cellular component of synaptosome-enriched and synaptosome-depleted proteins using ShinyGO. The number of proteins involved in each pathway as well as the fold enrichment are indicated. Note that while many synaptic proteins are detected in both fractions, enrichment analysis resolves their spatial restriction to the synaptic compartment. (**E**) Volcano plot of the same contrast as (**C**) but showing only the proteins annotated in SynGO. (**F**,**G**) SynGO GSEA of synaptosome-enriched and synaptosome-depleted proteins (see E) in terms of bar graph showing the top 20 overrepresented SynGO annotations of synaptosome-enriched proteins (**F**) or sunburst plots (**G**). Synaptosome-depleted proteins did not show significant overrepresentation in SynGO (see Supplemental Figure S7 for ShinyGO annotation). SynGO sunburst plots showing the overrepresentation of synaptosome-enriched and synaptosome-depleted proteins for cellular component in SynGO (**G**). For a complete list of proteins, see Supplemental File S2.

Although one whole-tissue lysate sample created higher CoV values for the group (Supplemental Figure S6), excluding this sample yielded results comparable to those including the sample. Therefore, we proceeded with a DEqMS analysis using all eight samples with age and sex as covariates, comparing whole-tissue lysate and synaptosome groups directly. Using a relatively stringent detection limit (2 peptides per protein, detected in 75% of samples per group), we confidently measured 5816 proteins detected in both whole-tissue lysate and synaptosome preparations (Figure 5A; Supplemental File S2), of which 1379 proteins were annotated as synaptic using the synaptic gene ontology online tool SynGO (SynGO release 1.3 [33]). While this represents a high degree of detection overlap, it is important to note that detection in the lysate reflects aggregate cellular abundance and does not imply information regarding synaptic localization or local enrichment. When using DIA for quantification, peptides need to be detected across samples. Some proteins that were confidently detected in the lysate sample were not confidently detected after synaptosome isolation (truly “lost” proteins; Supplemental File S2). These were a small fraction of the total amount (812/(812 + 5816) = 12.3%), of which 27 were annotated as synaptic in SynGO (Figure 5A). Gene set enrichment using the gene ontology tool ShinyGO [32] for these 812 proteins showed they belonged primarily to the nucleus (Supplemental Figure S7), which was expected based on the way synaptosomes were isolated (see Supplemental Protocol S2). We detected a relatively small fraction of 248 proteins (248/(248 + 5816) = 4.1%) that were exclusively detected in the synaptosome fraction, of which 33 were annotated as synaptic in SynGO. Gene set enrichment of these 248 proteins did not show overrepresentation for any cellular component term, suggesting these proteins were from diverse origin. Because we used stringent criteria for quantification (i.e., 2 peptides and presence in 75% of samples per group), we checked what the effect would be of lowering the detection limit to 1 peptide. From the 248 undetected proteins in the lysate, 180 proteins could then be identified, while 68 proteins were not detected at all in the lysate samples. To check whether this was just a matter of stochasticity, we plotted the frequency of peptides detected in the synaptosome fraction (Supplemental Figure S6). From these 68 proteins, 50 were detected with 2 peptides in the synaptosomes, and 18 with ≥ 3 peptides, indicating that this might well have been a matter of chance detection in the synaptosome fraction, similar to the many proteins detected only by 1 peptide in the lysate. Yet, a small set of proteins that was absent or expressed at very low levels in the lysate (0 to 1 peptide) seems highly enriched in the synaptosome fraction (detected with ≥ 4 peptides in synaptosomes; Supplemental Figure S6). Although gene set enrichment did not show overrepresentation in cellular component, these proteins represent a diverse group of molecules with (possible) roles in synaptic organization, signaling, and membrane dynamics. Several of these (e.g., GRIP2, GABRG3, SLC17A8) are established synaptic or neurotransmission-related proteins [39,40,41,42], as well as the AMPA receptor auxiliary subunit SHISA6, which is highly expressed in the dendrites of the hippocampus CA1 region [43]. All are present in the SynGO database, reinforcing the specificity of our synaptosome preparation. Others (PRKCQ, TYK2, LRRK1, PTPN13 and XPR1) are kinases, phosphatases, and phosphate regulators, which may regulate synaptic plasticity or local signaling cascades [44,45,46,47,48]. Proteins such as LRRK1, RAB27A, and SPIRE2 [49,50,51,52,53] suggest active vesicular trafficking, and cytoskeletal remodeling at the synapse. The presence of UNC5D, PDZRN3, PTPN13, and N4BP3 indicates potential involvement in synaptic function or in facilitating cell communication, as all have been implicated in neuronal migration [54,55,56,57]. The fact that these proteins were nearly undetectable in total lysates, despite the high sensitivity of the timsTOF HT, underscores the unique biological value of fractionation for resolving low-abundance proteins that are spatially restricted to the synaptic compartment.

Next, we checked to what extent curated synaptic (i.e., SynGO) proteins were not detected in either lysate or synaptosomes. From the entire SynGO database of 1789 curated protein entries [33], 339 proteins were not detected in either sample (‘SynGO absent’ proteins (cf. Figure 5A)). Gene set enrichment of this group of proteins indicated that among the highly overrepresented GO groups were acetylcholine receptor subunits, as well as receptor proteins from several families (Supplemental Figure S7, Supplemental File S2). The expression level of these proteins is very low, and levels of acetylcholine receptor subunits typically have been measured by receptor binding studies with subsequent immunoprecipitation [58,59].

From a quantitative perspective, our comparative LC-MS/MS analysis between whole-tissue lysate and synaptosome showed the significant (adjusted *p*-value < 0.01) enrichment of a relatively small set of proteins in synaptosomes (628 out of 5816 = 10.8%), and a marked depletion of a relatively large set of proteins (3278 out of 5816 = 56.4%) (Figure 5B,C). As expected, synaptic terms were enriched in proteins with a higher expression in synaptosomes, and nuclear and extracellular proteins were enriched for proteins with a higher expression in the whole-cell lysate samples (Figure 5C). From synapse-enriched and synapse-depleted proteins, 47.8% (300 out of 628) and 20.4% (668 out of 3278) were annotated as synaptic proteins, respectively (Figure 5E). Specifically focusing on the detected SynGO proteins in this contrast (Figure 5E; 300 enriched and 668 depleted), we observed enrichment of typical synaptic proteins, such as proteins of the vacuolar ATPase complex (e.g., ATP6V0A1) [60] and metabotropic GABA_B_ receptor subunits (e.g., GABBR2) [61], as well as synaptic depletion of heterogeneous nuclear ribonucleoproteins (e.g., HNRNPK and HNRNPL) [62]. Further gene set enrichment using ShinyGO [32] showed that proteins enriched in synaptosomes were predominantly associated with membrane components (ion channels, transporters) and synaptic compartments (Figure 5D). In contrast, proteins enriched in whole-tissue lysates (i.e., synaptosome-depleted) were overrepresented in categories such as cytosolic ribosomes, nuclear related components, and extracellular components (Figure 5D). A more in-depth examination of synapse-specific proteins using SynGO showed a significant overrepresentation in both presynaptic and postsynaptic compartments for the 300 synaptosome enriched SynGO proteins (Figure 5F,G). Although many SynGO proteins were detected in the synaptosome-depleted set, these 668 proteins did not show significant enrichment for cellular localization terms in SynGO (Figure 5G). GO annotations of this synapse-depleted SynGO protein set indicated predominant associations with ribosomal components and organelles of extracellular origin (Supplemental Figure S7), as required for local translation to meet the plasticity demand of the synapse [63,64]. Together, these proteins are not classical synaptic proteins (e.g., glutamate receptors), rather they are (multifunctional) proteins with multiple cellular localizations, one of which is the synapse.

Given the skewness of the volcano plot, we assessed the distribution of SynGO-annotated proteins (Supplemental Figure S8, and accompanying supplemental results) and concluded that the observed proteomic shifts are driven by genuine compartment-specific protein enrichment. This indicates that the normalization approach implemented in MS-DAP [30] is sufficiently robust to accommodate large differences between the sample types analyzed.

Lastly, we performed three comparative analyses with previous studies to verify our workflow: (1) correlation profiling of subcellular fractions from mouse hippocampus, cortex, and cerebellum [19]; (2) a synaptic proteome database from human, rat, and mouse based on 58 studies [65]; and (3) a cell-type-specific mouse synapse profiling study [4]. The mouse correlation profiling study of subcellular fractions [19] exhibited similar relative abundance patterns in the human samples, with a strong and statistically significant correlation (Pearson’s *r* = 0.64; Supplemental Figure S9 and accompanying supplemental results). While human proteins largely mirrored mouse localization in pre- and postsynaptic compartments, the depletion of specific neurofilaments [66,67] was attributed to their concentration in the postsynaptic density rather than technical variability. These findings underscore the biological conservation of synaptic protein distribution while highlighting specific divergence in organelle-associated protein regulation between species. The 58-study synaptic proteome database [65] revealed a high overlap with our synaptosome and lysate preparations (Supplemental Figure S10, and accompanying supplemental results). While the reference database contained a large number of unique protein IDs, functional enrichment analysis suggests these additional proteins are primarily nuclear contaminants rather than genuine synaptic components. This comparison confirms the high purity and comprehensive coverage of our optimized human brain proteomics workflow. Comparison with cell-type-specific mouse synapse proteomics [4] confirmed high coverage of synaptic markers in our dataset, with over 98% of excitatory, inhibitory, and common synaptic proteins detected (Supplemental Figure S11, and accompanying supplemental results). We observed that excitatory-enriched markers were significantly overrepresented in our synaptosome fraction compared to inhibitory or common markers. This shift suggests that the synaptosome-enriched proteome in the human cortex primarily reflects protein signatures from the abundant excitatory neuronal population.

## 4. Discussion and Conclusions

In this study, we first optimized our pre-mass spectrometry (pre-MS) workflow for neuroproteomic analysis with LC-MS/MS of post-mortem human gray matter (GM), and then used this optimized pipeline to compare whole-tissue lysate and synaptosome-enriched proteomes from human cortex. Post-mortem brain samples are typically received as frozen tissue blocks and are often processed for multiple downstream applications, including proteomics and immunohistochemistry. In this context, the separation of GM from white matter (WM) provides a critical advantage. GM is enriched in neuronal cell bodies and synaptic components [5], whereas WM contains abundant myelin proteins and oligodendrocyte-derived material [6,7]. Analyzing GM separately improves the detection of lower-abundant neuronal and synaptic proteins that could otherwise be masked by highly abundant myelin proteins, thereby increasing the biological specificity and interpretability of proteomic data.

Laser capture microdissection from thin tissue sections (5–10 µm) provides a precise approach to GM isolation [68,69], but its labor-intensive nature makes it impractical for large-scale proteomic studies (e.g., >200 samples). To balance specificity and scalability, we optimized a workflow based on free-hand dissection of GM from thin cryosections to generate whole-tissue lysates. We further demonstrated that elevated solubilization temperatures markedly enhanced protein extraction efficiency, underscoring the importance of optimizing tissue processing parameters for human brain samples.

### 4.1. Optimization of Tissue Solubilization and Digestion

Tissue solubilization is a critical preparatory step in any proteomic workflow, as it directly influences protein yield, integrity, and the breadth of proteome coverage. Two principal strategies are commonly employed for protein extraction: chemical and mechanical solubilization [18]. Chemical solubilization relies on the use of surfactants (e.g., SDS, Triton X-100, CHAPS) and chaotropic agents (e.g., urea, thiourea, guanidine hydrochloride) to denature and solubilize proteins, including membrane-associated and hydrophobic proteins that are typically underrepresented in proteomic datasets [70,71]. Mechanical disruption—such as tissue grinding or sonication—is often essential for subcellular proteomics to physically liberate organelles or synaptic compartments [8,72,73,74,75,76]. However, these multi-step mechanical procedures inherently introduce higher technical variability. In our study, we observed that while the chemical-only whole-tissue workflow maintained a low coefficient of variation (CoV ~15%), the synaptosome preparation—which necessitates mechanical homogenization in addition to ultracentrifugation—exhibited an increased CoV of ~22% (cf. Supplemmental Figure S6A). This suggests that while mechanical processing is indispensable for spatial proteomics, it probably comes at the cost of reduced technical reproducibility compared to streamlined chemical lysis. Thus, for large-scale studies where high throughput and low batch-to-batch variation are prioritized over spatial resolution, a chemical-only approach using well-defined surfactants (e.g., SDS) remains the more robust choice [71], particularly when working with small or heterogeneous human samples [2,72,73]. Furthermore, chemical solubilization facilitates scalability, though it requires the effective removal of MS-incompatible reagents, achieved here through the sTRAP method [3].

Here, we further optimized the sTRAP digestion protocol previously published by our lab using mouse brain tissue [3], employing an alternative chromatographic column that is more cost effective. The rationale for this optimization was to accommodate the specific requirements of human GM samples, including tissue quantity and protein input. First, we employed a high extraction temperature (95 °C) in a one-tube workflow, which benefits from a large surface area-to-volume ratio. While the original Protifi S-TRAP protocol recommends vortexing at room temperature or the inclusion of sonication or benzonase steps followed by reduction at 55 °C, our data demonstrate that elevated extraction temperature enhances protein solubilization and identification. This effect was likely further strengthened by using thin gray matter tissue sections, which facilitate more efficient heat transfer and extraction of insoluble proteins. Second, we substituted the vendor-recommended S-methyl methanethiosulfonate (MMTS) with 2-chloroacetamide (2-CAA). The use of 2-CAA enables complete and irreversible cysteine alkylation, rendering modified cysteines inert to TCEP and thereby improving reproducibility, peptide identification (cf. Table S2), and confidence in peptide–spectrum matches, while also simplifying database searching [35]. Importantly, 2-CAA is compatible with a wide range of buffers, including SDS, which we used here to achieve more efficient solubilization of brain tissue and membrane-associated proteins. These adaptations resulted in a highly standardized and efficient sample preparation workflow, which is an essential prerequisite for detecting subtle biological effects in human post-mortem tissue. Importantly, these optimizations did not lead to selective loss of specific protein groups, as evidenced by the low overlap among non-detected proteins across conditions. Together, these modifications facilitate the analysis of larger sample cohorts, thereby enhancing the scalability and applicability of the workflow.

Besides sTRAP, which offers a practical combination of affordability, straightforward design, and high reproducibility [74], numerous other sample preparation techniques have become available for proteomics (see Box 1), some of which were not published at the time of the experiments performed in the current study.

An important consideration throughout the entire experimental process that should be practiced independent of the use of rodent or human tissue as input material—including tissue dissection and LC-MS/MS analysis—is strict standardization and randomization [75]. This is especially important in large-scale studies where experiments are spread over extended periods. To mitigate confounding batch effects in the interpretation of the resulting data, we distributed the samples of each experimental group evenly across all experimental batches for every experimental step. In addition, we randomized the order of sample dissection, sTRAP preparation, and LC-MS/MS analysis to avoid confounding effects. Notably, we observed a trend in LC-MS/MS analysis where the order of samples ran affected the number of identified peptides (cf. Supplemental Figure S1). This underscores the critical role of rigorous standardization and randomization in large-scale proteomic workflows to ensure that technical variation affects all experimental groups equally. The workflow described here may serve as a practical guide for researchers aiming to standardize and refine neuroproteomic analyses from human brain tissue dissection through LC-MS/MS analysis.

In the present study using human brain samples, we confidently identified ~6000 to over 7400 proteins from human brain tissue, based on an initial detection of more than 82,000 to ~105,000 peptides (Figure 2 and Figure 5). This depth exceeds those reported in comparable studies analyzing whole-tissue lysates from human cortex or brain organoids [76,77,78], and approaches that of previous studies using pre-fractionation of isolated synaptosome samples (Table S1 [16]). Together, these results reflect recent advances in mass spectrometry sensitivity and support the suitability of chemically solubilized whole-tissue lysates for high-depth human neuroproteomics.

Box 1. **Alternative methods for proteomics sample preparation.**

NanoPOTS (nanodroplet processing in one pot for trace samples) performs all the proteomic processing steps (extraction, alkylation, digestion and peptide collection) in a semi-automatic manner within a single droplet, making this technique ideal for small biological samples (as low as 10–100 cells) as it reduces sample loss and increases robustness and reproducibility for high-throughput studies [79]. However, it requires highly specialized equipment and costly materials, making this technique less accessible.SP3 (single-pot, solid-phase-enhanced sample preparation) uses hydrophilic magnetic beads to capture proteins in a single tube, facilitating addition and removal of reagents needed for protein preparation for mass spectrometry and resulting in virtually no loss of material [80]. SP3 is compatible with a wide range of reagents and can be used for both low and high quantities of input material, but with protein amounts of >500 μg—as in the current study—the beads show aggregation, sticking to pipette tips and tube walls and resulting in protein loss.iST (in-StageTip) is performed in a single device as well, which is a pipette tip with a reaction chamber in which all preparation steps take place and a C18 filter at the bottom for final peptide cleanup [81,82]. It is a simple and robust method, but it shows incompatibility with detergents such as SDS that greatly improve cell lysis and protein yield [80].The One-Tip methodology is a more recent development, building upon the one-pot reaction [83]. Requiring only two pipetting steps, it combines a cell lysis and digestion buffer with the sample suspended in PBS into an Evotip (Evosep Biosystems) for pre-MS processing. It is suitable for single-cell analysis as well as for bulk proteomics from tissue sections.

### 4.2. Whole-Tissue Lysate Versus Synaptosome Proteomics

In the second part of this study, we directly compared human whole-tissue lysate and synaptosome-enriched samples to assess their relative strengths in detecting disease-relevant protein changes. We found that MS sensitivity was sufficient to detect most of the low-abundant (synaptic) proteins in whole-tissue lysates (cf. Figure 5, Supplemental Figure S7). We observed 1417 SynGO proteins in whole-tissue lysate (21.4% of proteome) and 1423 in isolated synaptosomes (23.5% of proteome). Moreover, virtually all SynGO proteins detected in our synaptosome fraction were also present in the whole-tissue lysate comparison (97.8%). These results suggest that for the broad detection of synaptic protein species, modern MS sensitivity significantly reduces the necessity for physical enrichment. In addition, synaptosome preparation is inherently more time consuming and labor intensive, which contributes to a higher between-sample variability. This may limit its practicality in large-scale studies investigating broader proteomic changes in disease. Importantly, detection overlap does not imply functional or regulatory equivalence. Therefore, subcellular fractionation retains clear biological value because it allows the detection of compartment-specific regulation that is likely masked in a whole-tissue context. Thus, combining lysate-level and synaptosome-level proteomics provides a more complete and biologically informative picture of synapse-specific processes and their modulation, revealing compartment-resolved regulatory mechanisms.

Based on these considerations, we propose whole-tissue lysate proteomics as an effective first-line strategy to identify disease-associated proteomic changes. This approach offers a broad, rapid, and technically robust overview of molecular changes without compromising the depth of (low-abundant) synaptic protein detection. Whole-tissue lysate analysis is particularly advantageous when tissue is collected in parallel for both whole-tissue lysate and synaptosome preparations as described here. In such cases, initial whole-tissue lysate data can highlight key biological processes or pathways that may warrant deeper, targeted (synapse-focused) investigation through synaptosome proteomics to resolve local protein redistribution or stability.

### 4.3. Synaptosome Isolation and Interpretation

Synaptosomes in this study were isolated using a sucrose density gradient [26], a method with which our laboratory has extensive experience for synaptosome, synaptic membrane, and post-synapse enrichment [19,24,84,85,86]. Alternative gradient-based approaches, including Ficoll (sucrose and epichlorohydrin copolymer [26,87]) and Percoll (colloidal silica particles covered in polyvinylpyrrolidone monolayer [88]), are also commonly used. While these methods share similar principles, sucrose density gradients have been shown to result in lower contamination by extrasynaptic mitochondria than Ficoll gradients [89]. Although Percoll gradients offer increased speed [88], sucrose gradients yield a higher proportion of viable synaptosomes and preserve synaptic vesicles more effectively [90], while also reducing mitochondrial contamination compared to Ficoll gradients [89]. Together, these considerations support the choice of sucrose density gradient fractionation for the present study.

It is important to emphasize that biochemical fractionation enriches rather than purifies subcellular compartments and therefore carries an inherent risk of co-isolating non-synaptic proteins. A limitation of our study is the lack of independent assessment of synaptosome purity or structural integrity (e.g., by electron microscopy), precluding an absolute benchmark of structural integrity. However, this method has previously been validated in our laboratory ([24]; Dr. Rao-Ruiz, personal communication). Residual contamination from non-synaptic membranes and organelles is therefore unavoidable and should be interpreted accordingly [9,91], as also reflected by the presence of nuclear contaminants in the synaptic proteome meta-analysis (cf. Supplemental Figure S10; [65]).

Despite these limitations, multiple lines of evidence support the biological relevance of our synaptosome-enriched dataset. Annotated synaptic proteins (SynGO; [33]) were significantly enriched in the synaptosome fraction and showed strong overrepresentation in gene ontology pathways associated with both presynaptic and postsynaptic compartments (cf. Figure 5). Proteins relatively depleted from the synaptosome fraction did not exhibit enrichment for synapse-specific pathways, and proteins absent from synaptosomes were predominantly nuclear—consistent with their removal during early fractionation steps. Using our optimized workflow, we achieved high proteomic coverage, identifying over 6600 proteins in whole-tissue lysates, which favorably compared to the coverage reported in comparable human studies [8,69,92]. 

In synaptosome-enriched samples, we identified and quantified over 6000 proteins. Although this depth is comparable to the 2019 study of Alzheimer disease donors by Hesse et al. (Table S1; [17]), only 440 (8.2% of proteome) of these were annotated as SynGO proteins. Several factors may contribute to this relatively low proportion. First, methodological differences in synaptic enrichment—such as the use of crude P2 fractions instead of gradient-purified synaptosomes—can substantially affect synaptic specificity. Second, disease-related synapse loss or protein depletion may selectively reduce the detectability of synaptic proteins in neurodegenerative cohorts. Indeed, in the study by Plum et al. [15], which examined synaptosome proteomes from Parkinson disease and control donors (Table S1), SynGO coverage reached 21% overall, but this increased to 42.2% when considering control donors alone. In contrast, Kandigian et al. [93] reported similar SynGO coverage in control (35.4%) and Alzheimer disease (33.7%) samples (Table S1), using differential (ultra)centrifugation without gradient purification.

Importantly, higher proportions of SynGO proteins are often observed in studies with more limited overall proteome coverage (Table S1), where the most abundant and well-annotated synaptic proteins are preferentially detected, see for example the study of Kumar et al. [94]. Conversely, studies achieving very deep proteome coverage tend to report lower relative SynGO percentages. This is exemplified by the large-scale synaptic proteomics study by Aryal et al. (>7200 quantified proteins; Table S1 [16]), in which SynGO proteins accounted for only 19.5% of the dataset; very similar to what we reached here (23.5%). Together, these comparisons suggest that the proportion of SynGO-annotated proteins is strongly influenced by both the depth of proteome coverage and the specific enrichment strategy used, rather than serving as a simple indicator of synaptic specificity.

### 4.4. Proteome Coverage and Future Directions

Despite the depth of proteome coverage reached, our data also highlight current limitations in human brain proteomics. Transcriptomic analyses indicate that more than 14,000 protein-coding genes are expressed in the human cortex [95,96], implying that a substantial fraction of the proteome remains undetected. This is likely due to the complexity of human brain tissue and the current sensitivity limits of LC-MS/MS. Strategies to reduce sample complexity, such as optimized acquisition modes, physicochemical fractionation, or subcellular enrichment [93,97,98], as well as deeper proteomic approaches using multiple proteases and fragmentation methods [16,99,100], may further expand coverage in future studies.

On the other hand, increased MS sensitivity also raises the likelihood of detecting non-synaptic proteins in synaptosome preparations [65] (cf. Supplemental Figure S10), complicating interpretation in human tissue where genetic labeling strategies are not available [101].

### 4.5. Conclusions and Recommended Strategy

While our results demonstrate that modern LC-MS/MS provides synaptic protein coverage in whole-tissue lysates largely comparable to that of synaptosome-enriched samples (Figure 5), this approach serves as a complement to, rather than a substitute for, subcellular fractionation. Subtle or compartment-specific protein regulation—such as altered local stability or transport—may be obscured in whole-tissue analyses, particularly for proteins with multiple cellular localizations. Conversely, while synaptosome proteomics provides a unique window of insight into the spatial regulation and composition of the synapse, it introduces challenges related to normalization and interpretation when synapse density itself is altered, as often seen in neurodegenerative conditions [11,102]. In such contexts, the whole-tissue proteome serves as a critical reference point, allowing researchers to distinguish between localized protein redistribution and an overall shift in synaptic abundance.

Therefore, we recommend a tiered neuroproteomic strategy: initial whole-tissue lysate analysis to identify broad disease-associated processes, followed by targeted synaptosome proteomics to resolve specific synaptic alterations. By utilizing the same set of donor samples for both layers of analysis, this combined framework maximizes sensitivity and interpretability across cellular and subcellular levels. Ultimately, this integrated approach respects the biological importance of spatial resolution while maintaining the high-throughput reproducibility required for large-scale human neuroproteomics studies.

## Figures and Tables

**Table 1:** Demographic and clinical information of the superior frontal gyrus samples used in subcellular fraction isolation (synaptosome proteomics). NDC = non-demented control; NA = not measured; Braak LB = Braak Lewy bodies; PMI = post-mortem interval; APOE = apolipoprotein E allele.

NDC Donors (*n* = 8)	
Female/male *n*	4/4
Age median (range)	88 (83–93)
Braak median (range)	2 (0–3)
Braak LB (*n*)	0 (1), 2 (1), 3 (1), NA (5)
PMI median (range)	06:48 (05:05–10:00)
pH median (range)	6.67 (6.20–7.12)
APOE (*n*)	3-3 (1), NA (7)

## Data Availability

Raw proteomics data are available via ProteomeXchange with identifier PXD076278. The processed data presented in this study are included in the supplementary material. Further inquiries can be directed to the corresponding author.

## Supplementary Materials

The following supporting information can be downloaded at https://www.mdpi.com/article/10.3390/cells15080736/s1; Table S1: Overview of human proteomics studies focusing on the synapse; Table S2: Impact of tissue extraction temperature on the efficiency of cysteine reduction and alkylation; Table S3: Number of peptides with missed trypsin cleavage sites from trypsin pilot experiment; Figure S1: Necessity of randomization in MS workflow; Figure S2: Determining isolated protein amount by BCA test; Figure S3: Number of peptides/proteins detected in pilot; Figure S4: Solubilization at 95 °C increased the number of peptides per protein; Figure S5: (De)enrichment of proteins by centrifugation is minimal; Figure S6: Variation and differentially detected proteins in whole-tissue lysate and synaptosome samples; Figure S7: Protein pathways of lost synaptosome proteins and absent SynGO proteins; Figure S8: Binning of differential expression values indicates correct normalization between synaptosome and lysate samples; Figure S9: Cross-validation of synaptosome enrichment and depletion; Figure S10: Comparison with a meta-analysis of unique synaptic proteins (based on Sorokina et al. [65]); Figure S11: Distribution of proteins related to inhibitory and excitatory cell types (based on Van Oostrum et al. dataset [4]); Supplementary File S1: Differential abundance protocol optimization; Supplementary File S2: NDC lysate vs. synaptosomes; Supplementary Protocol S1: Pre-MS workflow; Supplementary Protocol S2: Synaptosome isolation.

## Abbreviations

The following abbreviations are used in this manuscript:

LC-MS/MS	Liquid Chromatography-Tandem Mass Spectrometry
sTRAP	Suspension Trapping
NBB	Netherlands Brain Bank
SFG	Superior Frontal Gyrus
NDC	Non-Demented Control
AD	Alzheimer’s Disease
GM	Gray Matter
PMI	Post-Mortem Interval
Braak LB	Braak Lewy Bodies
APOE	Apolipoprotein E
SDS	Sodium Dodecyl Sulfate
BCA	Bicinchoninic Acid
DEqMS	Robust statistical inference for quantitative LC-MS proteomics
DIA-NN	Data-Independent Acquisition—Neural Networks
dia-PASEF	Data-Independent Acquisition—Parallel Accumulation Serial Fragmentation
VSN	Variance Stabilizing Normalization
MS-DAP	Mass Spectrometry Downstream Analysis Pipeline
FDR	False Discovery Rate
SynGO	Synaptic Gene Ontology
ShinyGO	Shiny Gene Ontology
DIA	Data-Independent Acquisition
SNARE	Soluble NSF Attachment Receptor
GO	Gene Ontology
CHAPS	3-[(3-cholamidopropyl)dimethylammonio]-1-propanesulfonate
WM	White Matter
