# Supplementary material for: A Tiered Approach to Human Synapse Proteomics: Optimized LC-MS/MS Analysis of Whole-Tissue Lysate and Synaptosome Preparations from Frozen Post-Mortem Brain Samples
Source: Cells. 2026 Apr 21;15(8):736. doi: 10.3390/cells15080736 (PMC13114917; doi:10.3390/cells15080736)
Supplement: Supplementary file 1 [file cells-15-00736-s001.zip › cells-4164439-Supplemental tables and figures.pdf]

Supplemental tables and figures:

Table S1. Overview of human proteomics studies focusing on the synapse, using similar cut-off for peptide quantification as used in the present study for comparison.

**Tissue type, *n*-number**	**Tissue/peptide input**	**Synaptosome preparation**	**Proteins identified & quantified**	**Number SynGO proteins of total (% proteome); data taken from their Supplemental Tables:**	**Authors**
Prefrontal cortex, BA10); 5 AD and 5 control donors measured in duplicate	50 mg tissue	Syn-PER Reagent isolation	386 quantified (2 peptides in 80% of each sample group); 30 proteins with unavailable refseq numbers	180 from 356 proteins (50.6%); Table S9	Kumar et al. 2025 [94]
DLPFC (BA46), 34 Schizophrenia, 34 Bipolar disease, 35 control donors	14 µg peptides for TMT	Sucrose gradient for synaptosomes	8329 identified (≥ 2 peptides) with single gene symbol; 7217 with ≥70% of all samples detected with single gene symbol	1410 of 7217 proteins (19.5%); Tabel S2	Aryal et al. 2023 [16]
Angular gyrus sections; 5 AD, 5 control donors	200 mg tissue	Differential centrifugation, e.g., P6 (large protein complexes), S6 (cytosol)	2211–2223 proteins identified in either P5 or P6 samples of controls (≥ 2 peptides in at least 80% of each sample); 1978 proteins shared between P5 and P6 in controls	700 of 1978 proteins (35.4%); Supplementary Table 2	Kandigian et al. 2022 [93]
Substantia nigra, 5 Parkinson, 5 control donors	NA (200 ng peptides loaded)	Percoll gradient for synaptosomes	2764 proteins identified with single gene symbol; 801 quantified (2 peptides in 80% of each sample group) with single gene symbol	168 of 801 proteins (21.0%); Supplemental Table S3	Plum et al. 2020 [15]
Superior temporal gyrus BA41/42) & primary visual cortex (BA17), 33 donors	300 mg (25 µg peptides for TMT)	P2 fraction derived from homogenate	5421 identified (≥ 2 peptides); 5348 in ≥75% sample pair groups with single gene symbol	440 of 5348 proteins (8.2%); Table S5	Hesse et al., 2019 [17]

Table S2. Impact of tissue extraction temperature on the efficiency of cysteine reduction and alkylation. The average number of precursors identified consistently (75% of technical replicas) within the samples belonging to each incubation condition per donor type, both in total and when containing a modified (carbamidomethylated) cysteine residue.

**Extraction temperature**	**Control samples**		**AD samples**	
	**Total precursors**	**Precursors with a modified cysteine residue**	**Total precursors**	**Precursors with a modified cysteine residue**
**55 °C**	54,382	5561 (10.2%)	54,248	6439 (11.9%)
**95 °C**	56,803	9024 (15.9%)	55,814	8886 (15.9%)

Table S3. Number of peptides with missed trypsin cleavage sites from trypsin-pilot experiment. The number (%) of peptides with missed Arg (R) and Lys (K) sites from the total number of peptides analyzed (total peptides), as well as from the number of peptides that were present in all samples (i.e., quantifiable).

**Type of peptides**	**Total peptides**	**R-missed**	**K-missed (%)**	**Total missed**
**Total analyzed peptides**	79,586	4394 (5.5%)	4903 (6.2%)	9297 (11.7%)
**Quantifiable peptides (measured in all 12 samples)**	49,629	2059 (4.1%)	2401 (4.8%)	4460 (9.0%)

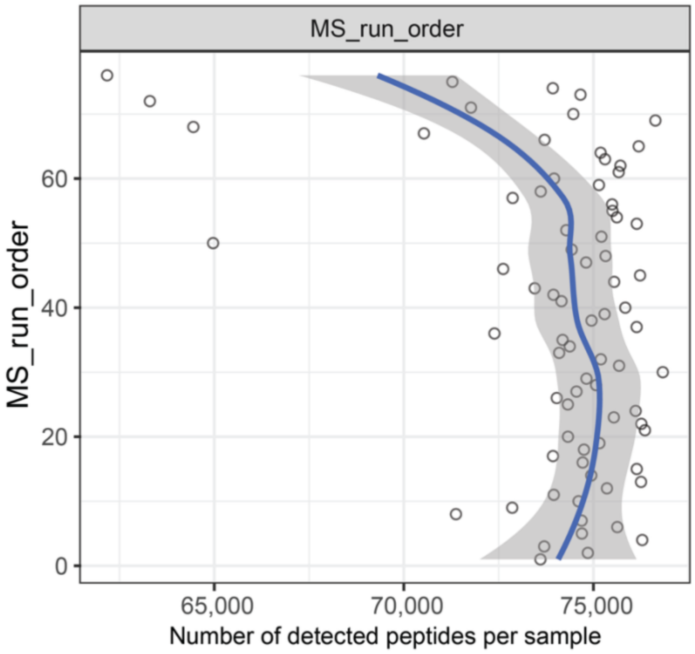

Figure S1. Necessity of randomization in MS workflow. One of the standard quality controls from the MS-DAP analysis shows the effect of the order in the MS analysis on the number of peptides detected. This emphasizes the importance of randomizing the samples across experimental groups to avoid technical effects biasing the results.

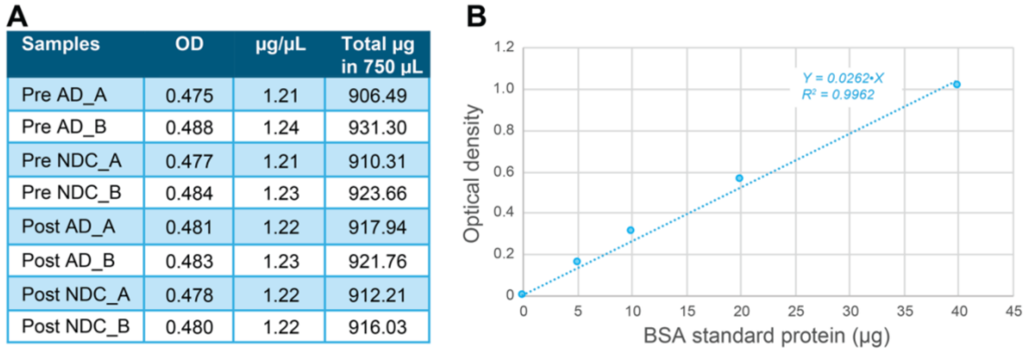

A

Samples	OD	μg/μL	Total μg in 750 μL
Pre AD_A	0.475	1.21	906.49
Pre AD_B	0.488	1.24	931.30
Pre NDC_A	0.477	1.21	910.31
Pre NDC_B	0.484	1.23	923.66
Post AD_A	0.481	1.22	917.94
Post AD_B	0.483	1.23	921.76
Post NDC_A	0.478	1.22	912.21
Post NDC_B	0.480	1.22	916.03

Figure S2. Determining isolated protein amount by BCA test. Optical density (OD) was measured for bovine serum albumin (BSA) standards (left) and was used to extrapolate protein concentration based on the OD of 15 μL from our samples (right). On average, our samples contained around 900 μg protein in 10 mg of initial tissue weight (dissolved in 750 μL 5% SDS for the OD measurements above). No weight loss was encountered by centrifugation. Pre = pre-centrifugation; Post = post-centrifugation; NDC = non-demented control donor; AD = donor with Alzheimer disease.

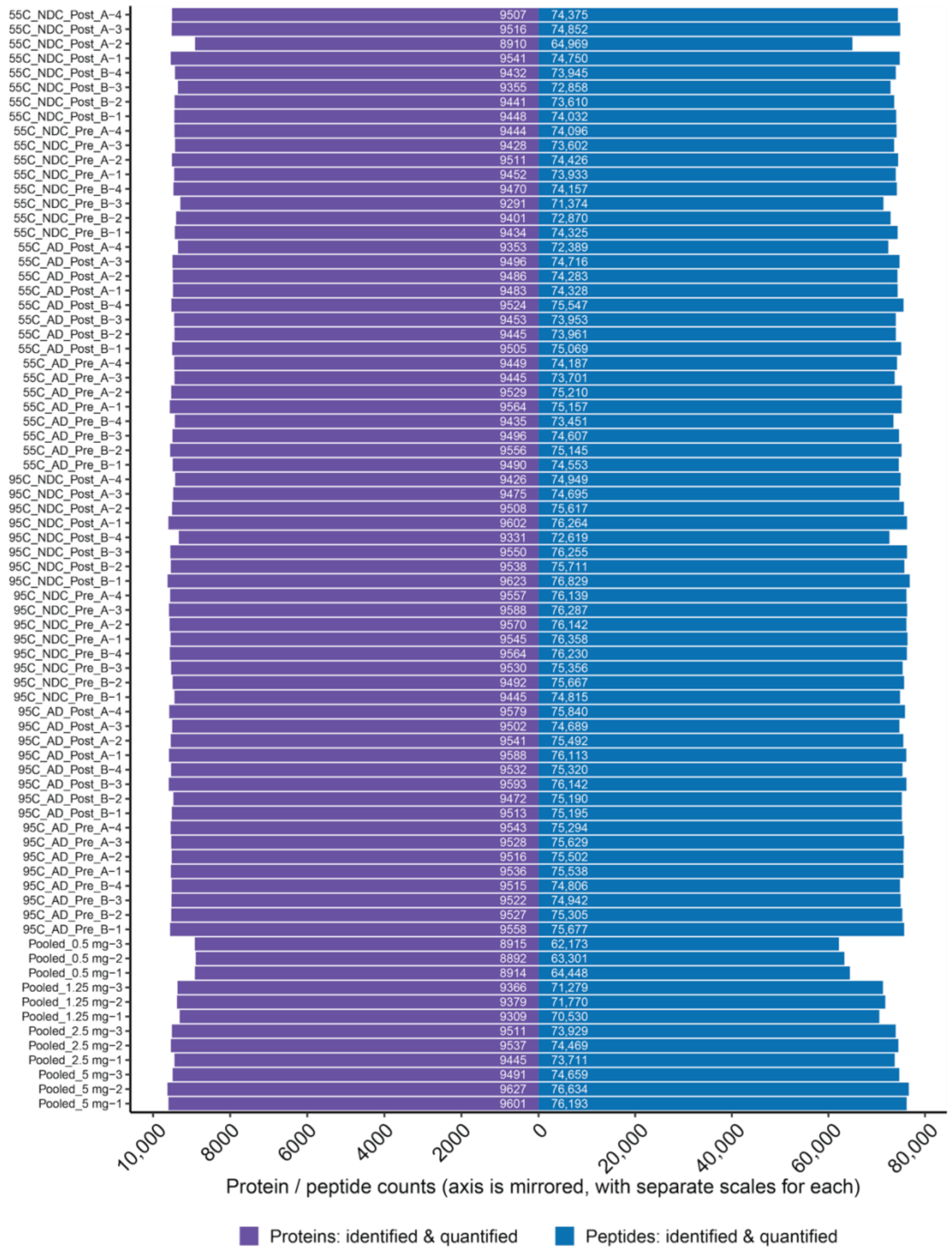

Figure S3. Number of peptides/proteins detected in pilot. This plot shows the number of (target) peptides that are detected per sample, as well as total protein number. For DIA, we refer to a peptide as 'detected' if the confidence score (for identification) is <0.01.

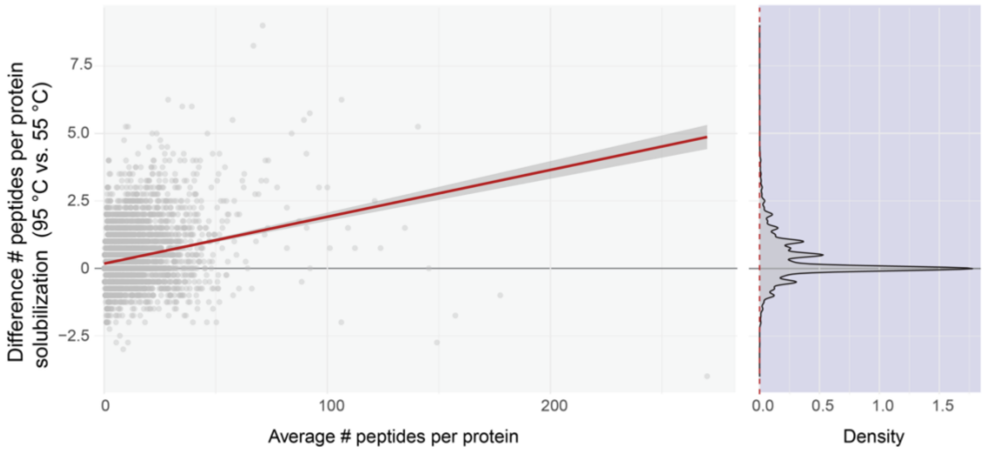

Figure S4. Solubilization at 95 °C increased the number of peptides per protein. *Left*; MA-plot for the difference in peptides per protein participating in the 95 °C contrasts (average of 4 contrasts) vs. those in the 55 °C contrast (average of 4 contrasts) (y-axis), and the average number of peptides per protein for all contrasts participating (x-axis, Log_{10} scale). The positive trend line and equation are shown. *Right*; Density plot of the difference in peptides per protein participating in the 95 °C contrasts vs. those in the 55 °C contrasts (y-axis). Together, this shows that most proteins increased the number of detected peptides at the 95 °C solubilization step. For data, see Supplemental file S1.

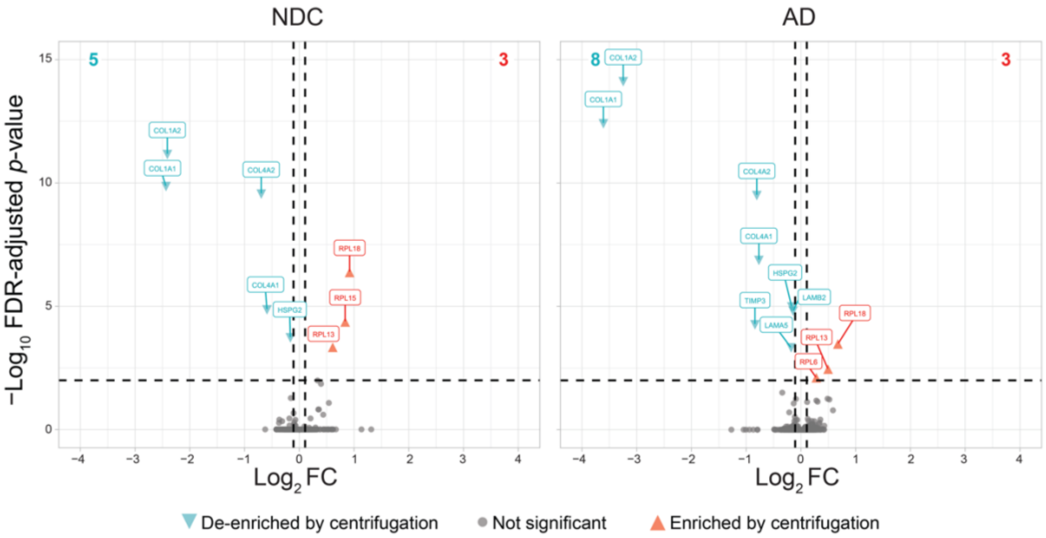

Figure S5. (De)enrichment of proteins by centrifugation is minimal. Volcano plots showing the DEqMS contrast of pre- vs. post-centrifugation for samples lysed at 55 °C, for control samples (NDC) and AD samples (AD). Gene symbols are shown for the significant proteins that are centrifugation-depleted (turquoise) or centrifugation-enriched (red) for adjusted *p*-values <0.01 (horizontal dashed line). The total number of differentially expressed proteins (colored; vertical dashed line; >|±0.10|) is indicated at the top (left, turquoise; right, red). Among the centrifugation-depleted proteins are multiple collagens. Proteins that are enriched by centrifugation include ribosomal proteins. Overall, the protein abundance decrease due to centrifugation is minimal, and greatly similar between the NDC and AD samples. The proteins for which no peptides were detected (truly "lost" proteins after centrifugation; not visible in this graph) were only a very small fraction of the total; Supplemental file S1.

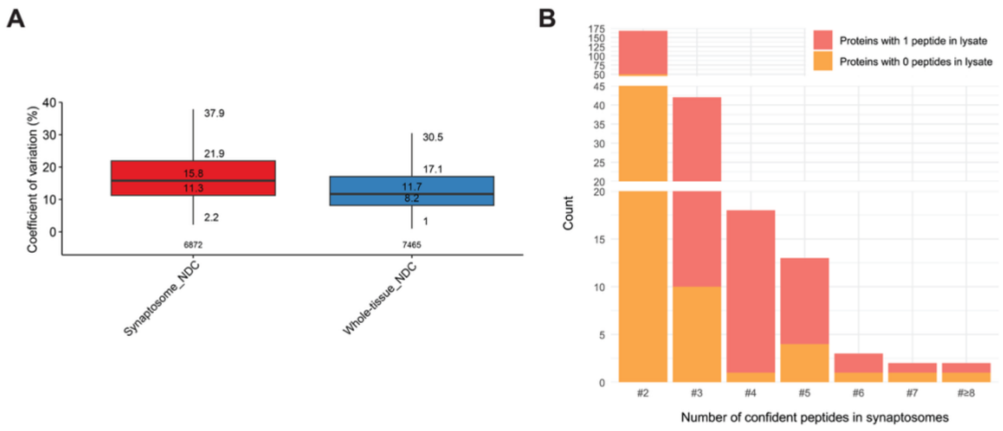

Figure S6. Variation and differentially detected proteins in whole-tissue lysate and synaptosome samples. (A) Coefficient of variation in protein samples of whole-tissue lysates and synaptosomes isolated from 8 NDC brains. Note that 1 whole-tissue lysate sample increased the protein CoV from 15.2 % to the current 17.1%. Due to marginal differences in protein regulation and detection, we kept the sample in. (B) Histogram of protein count detected in the lysate with 0 or 1 peptide (lost in lysate) by their peptide count detected in the synaptosome fraction. Proteins detected by 1 peptide in the lysate (n = 180) form the majority of these "lost" peptides and hence likely depend on stochastic events. Peptides confidently detected in synaptosomes from 4 and up (n = 38) form a group that are low expressed in lysate and highly enriched in synaptosomes.

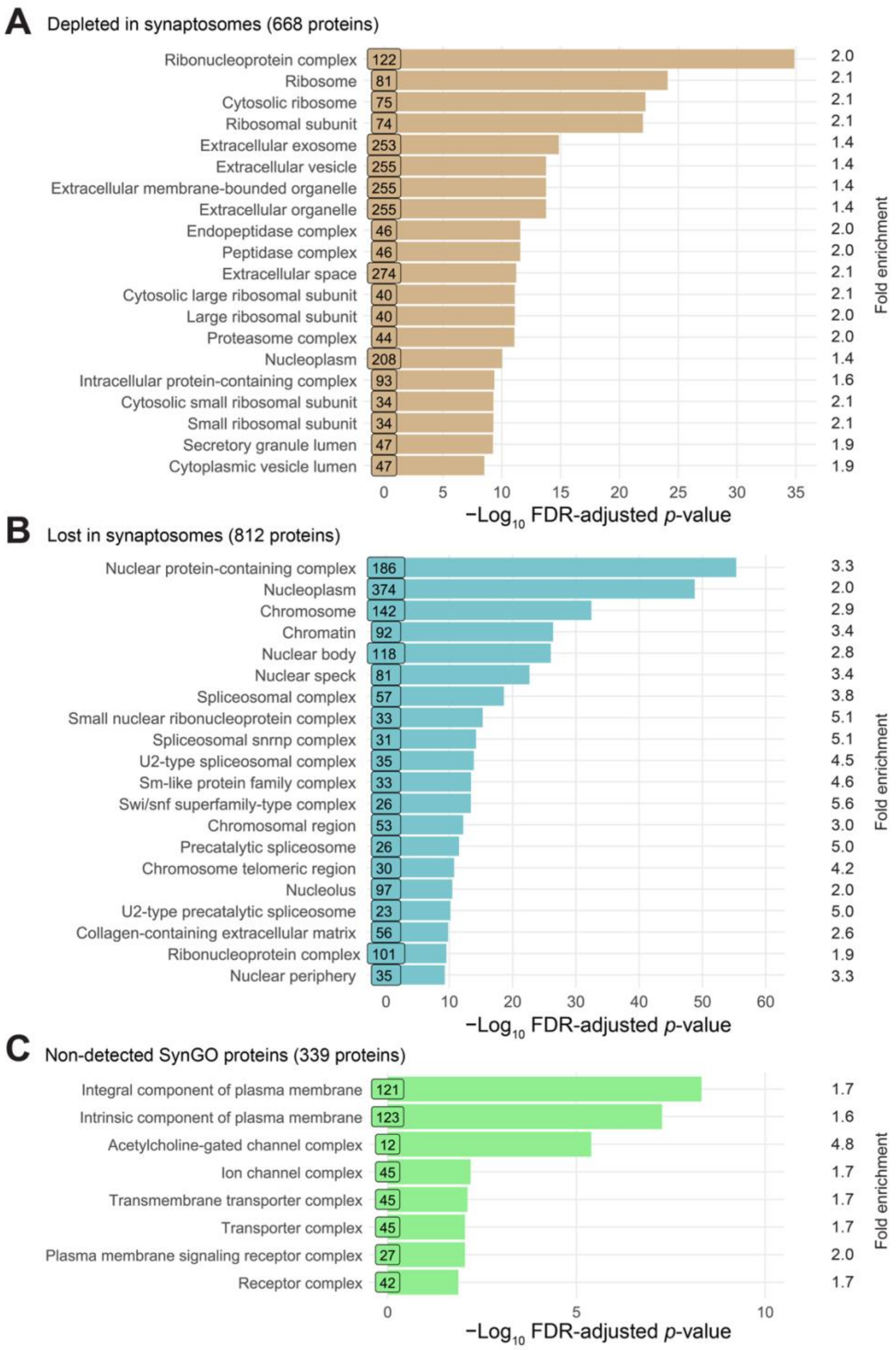

Figure S7. Protein pathways (cellular component, CC) of lost synaptosome proteins and absent SynGO proteins. (A,B,C) Bar graphs (ShinyGO) showing the top 20 overrepresented CC-GO annotations of the 668 SynGO proteins that were depleted in the synaptosome isolation; cf. Fig. 5A,E (A), the top 20 overrepresented CC-GO annotations of the 812 proteins that were not detected ("lost") in the synaptosome isolation; cf. Fig. 5A (B), and the 339 CC-SynGO proteins from the SynGO database that were not detected in either of the samples according to the criteria set; cf. Fig. 5A (C). Note that the x-axis scale is different for panels A–C. For a complete list of proteins in each analysis, see Supplemental file S2.

Supplemental results accompanying Figures S8 to S11.

Comparing whole-lysate and synaptosome samples is inherently challenging, as the different nature of these preparations may complicate normalization and raise concerns about potential bias, as reflected by the skewness of the volcano plot. To address this, we analyzed the distribution of SynGO-annotated proteins across 50-protein bins ordered by log_2 fold change (Supplemental Figure S8). Given the high number of SynGO proteins in bins 1–6, the remainder of the proteome was divided into three additional bins of comparable size, plus a final residual bin. Gene set enrichment analysis (GSEA) of SynGO terms revealed a clear overrepresentation in bins 1–6 and, to a lesser extent, in bins 7–18, the latter approaching the threshold for differential expression. Based on these results, we conclude that the observed proteomic shifts are driven by genuine compartment-specific protein enrichment. Furthermore, the normalization approach implemented in MS-DAP [30] is sufficiently robust to accommodate large differences between the sample types analyzed.

Previous correlation profiling of subcellular fractions from mouse hippocampus, cortex, and cerebellum—including microsomes, synaptosomes, synaptic membranes, and postsynaptic densities—revealed consistent patterns of functional grouping and subcellular localization [19]. Based on these findings, we selected sets of mouse proteins that were previously identified as either enriched (n = 192) or depleted (n = 134) in synaptosomes and synaptic membranes from the cortex. Of these, we detected 79 synapse-enriched and 117 synapse-depleted orthologs in our human cortex proteomics dataset. Notably, many of these proteins exhibited similar relative abundance patterns in the human samples, with a strong and statistically significant correlation between mouse and human data (Pearson r = 0.64, p < 0.001; Supplemental Figure S9A). Among the human proteins that mirrored the synaptic enrichment or depletion observed in mice (79 and 117 proteins, respectively), functional annotation revealed localization to either pre- and post-synaptic compartments (synapse-enriched) or to cytosolic, ribosomal, or membrane-associated structures (synapse-depleted) (Supplemental Figure S9B,C), similar to the previous analysis shown in Figure 5 and Supplemental Figure S7. Three notable subsets among the synapse-depleted proteins were the relatively high enrichment of the postsynaptic (intermediate filament) cytoskeleton and neurofilament groups, which included proteins like neurofilament medium chain (NEFM), and neurofilament light chain (NEFL) (see Supplemental file 2). They are known to function in the postsynaptic compartment [66,67], and are indeed enriched in the postsynaptic density fraction, but not in the synapse fractions [19]. We found these cytoskeletal proteins consistently identified with high confidence, each supported by more than 49 unique peptides, and they were equally detected across both sample types. Hence, the observed synaptosome-depletion stems from differences in relative abundance and subcellular localization (post-synaptic density-enriched) rather than technical variability. Surprisingly, a part of the proteins that were enriched in the synaptosome fraction in mice showed opposite regulation in our human dataset (n = 54). Gene ontology analysis showed that these proteins belonged to vesicle pathways and the Golgi apparatus (Supplemental Figure S9D).

Next, we compared our data with those of a meta-analysis of the synaptic proteome, covering 8044 unique protein IDs detected from 58 published synaptic proteomic datasets derived from different parts of the human, mouse and rat brain by Sorokina et al. [65] (Supplemental Figure S10A). Overlap with our synaptosome dataset was slightly higher (5096 proteins, 84.0%) than that for the lysate dataset (5414 proteins, 81.7%). SynGO

proteins were represented at a similar rate in the Sorokina dataset (1522, 18.9%), compared to that of the lysate (1298, 19.6%), and were both at a slightly lower rate than the synaptosome (1302, 21.5%) samples. Remarkably, the number of proteins that were unique to the Sorokina dataset was significantly higher (2363, 29.4%) than that for the synaptosome (109, 1.8%) and lysate (357, 5.4%) set. This could reflect biological variation due to use of data from different species and brain regions. However, a ShinyGo overrepresentation analysis of the 2363 unique proteins indicated a large proportion of proteins related to the nucleus (chromosome/chromatin, nucleosome) (Supplemental Figure S10B). This suggests that, at least in part, contamination due to the type of (biochemical) isolation used in the different studies of this meta-analysis contributed to annotating these proteins as synaptic.

Lastly, we compared our data to a proteomics dataset where the authors did not only isolate synaptosomes but also used fluorescent labeling in transgenic mice to find unique synapse-type-enriched proteins [4]. To ensure we selected synaptic proteins from the cortex, we took those that showed a significant enrichment for synapsin1 after sorting [4]; this resulted in 533 proteins (Supplemental file 2). Subsequently, we divided these proteins based on the enrichment of the Camk2 vs. GAD labeling, as being excitatory-enriched (E > I; 60, 11.3%), inhibitory-enriched (I > E; 67, 12.6%), or common (E = I; 406, 76.2%) (Figure S11A). As the authors mentioned [4], these proteins show a high percentage of SynGO annotations, and this is specifically true for the excitatory-enriched proteins (E > I; 46, 76.7%; Figure S11A). From the 533 synaptic proteins, we found a high coverage in our samples, with 523 detected in the lysate and 524 detected in the synaptosome samples, and 523 proteins in overlap (quantified). Inhibitory-enriched as well as common marker proteins show a distribution of synaptosome-enriched, -depleted, and non-regulated proteins similar to that observed in the total population (Figure S11B; cf. Figure 5C), albeit with a slightly higher proportion of synaptosome-enriched proteins at the expense of non-regulated proteins. Remarkably, the excitatory-enriched marker proteins show a significant increase (Chi-2 (12, n = 581) = 25.6, $p < 0.001$) in synaptosome-enriched proteins vs total proteome markers, mostly at the expense of synaptosome-depleted proteins (Figure S10B). This indicates that our synaptosome-enriched proteins largely stem from excitatory neurons, which are abundantly present in the cortex.

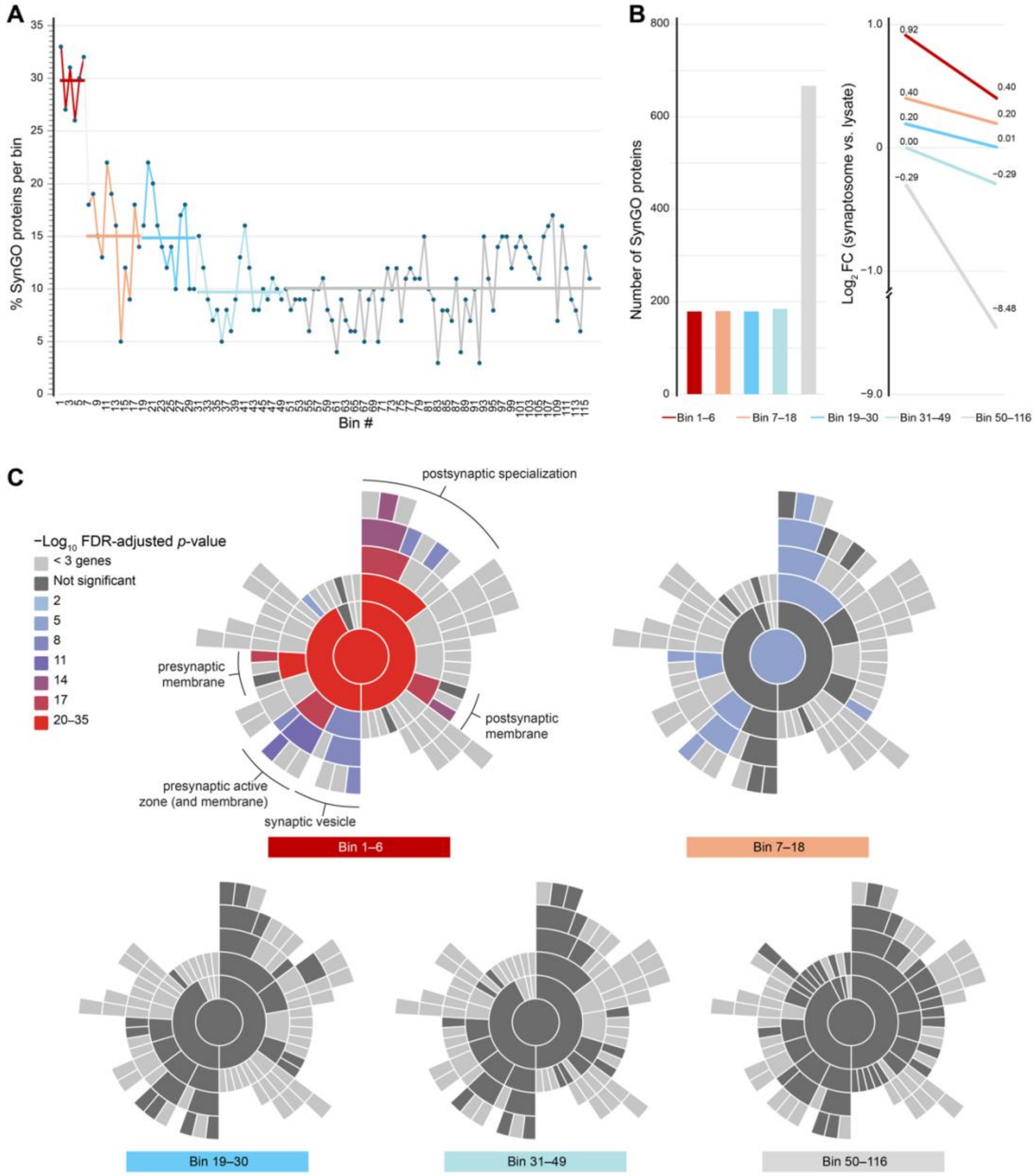

Figure S8. Binning of differential expression values indicates correct normalization between synaptosome and lysate samples. (A) Plot showing the percentage of SynGO proteins among the 116 bins (50 proteins) based on differential expression between synaptosomes and whole-cell lysate samples (log2FC). Based on this, 5 main groups (color coded) were detected, the first showing the largest group of SynGO proteins. (B) Bar graphs showing the total number of proteins per color-coded bin group (left) and the log2FC regulation values of each bin group (right). (C) SynGO GSEA sunburst plots showing the overrepresentation analysis mainly in bins1–6 and 7–18 when compared to the total proteome detected.

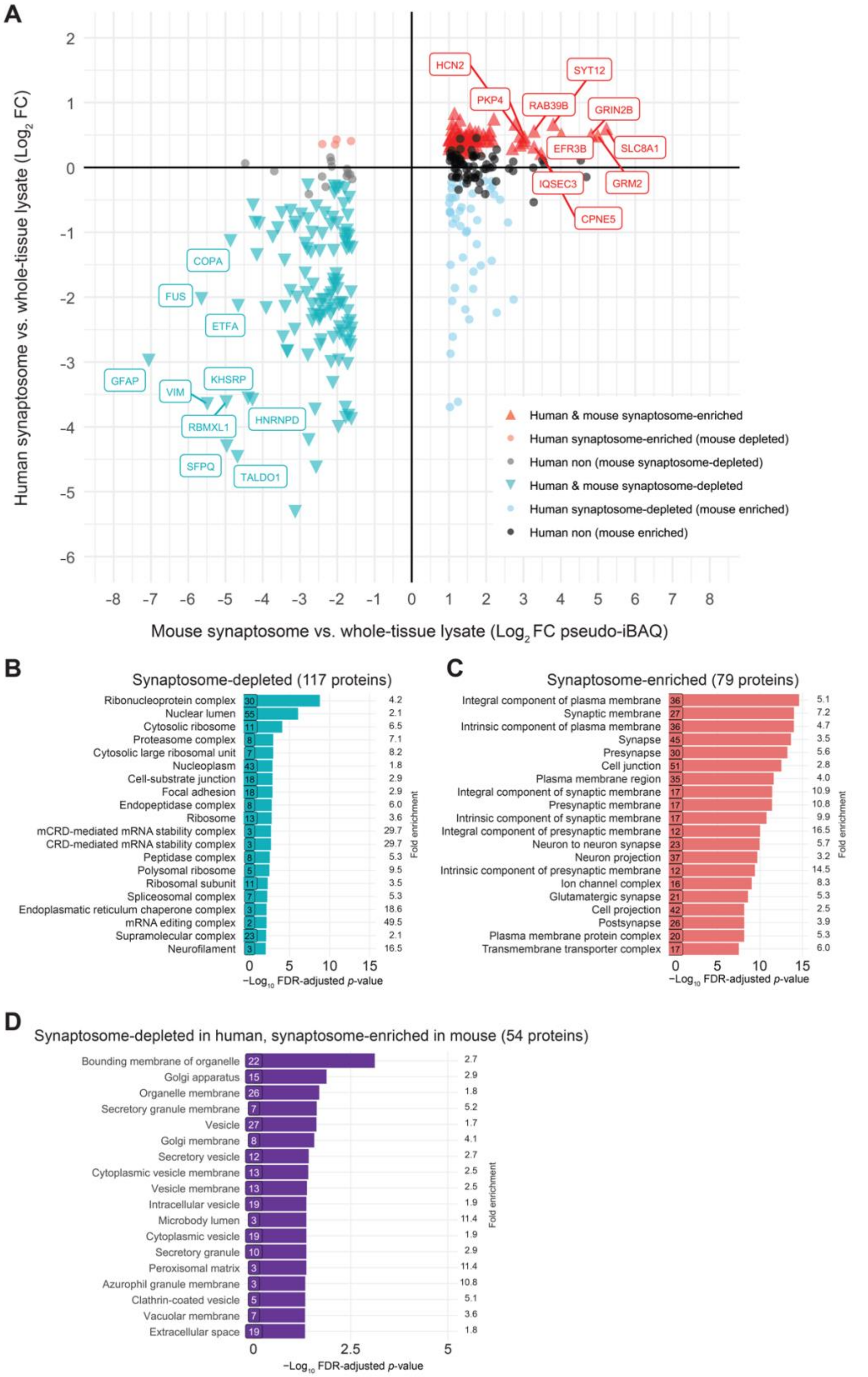

Figure S9. Cross-validation of synaptosome enrichment and depletion. (A) Using a previously published dataset of mouse cortical synapse-enriched and depleted proteins by differential biochemical fractionation [19], selected proteins (n = 326) were retrieved from our human NDC lysate vs. synaptosome data set, and their relative ratios (log2) were compared. The top 10 proteins with the highest enrichment in both mouse and human synapses are labeled. (B,C) Bar graph

showing the top 20 overrepresented GO annotations of mouse synaptosome (pseudo-iBAQ values) and human synaptosome proteins that were both depleted in synaptosomes (B) and enriched in synaptosomes (C) using ShinyGO. The number of proteins participating in each annotation, as well as the fold enrichment, are indicated. (D) Bar graph showing the 18 overrepresented cellular compartment CC-GO annotations (ShinyGO) of the 54 proteins that were enriched in synaptosomes in mouse [19] but were depleted in human synaptosomes. The number of proteins participating in each annotation, as well as the fold enrichment, are indicated.

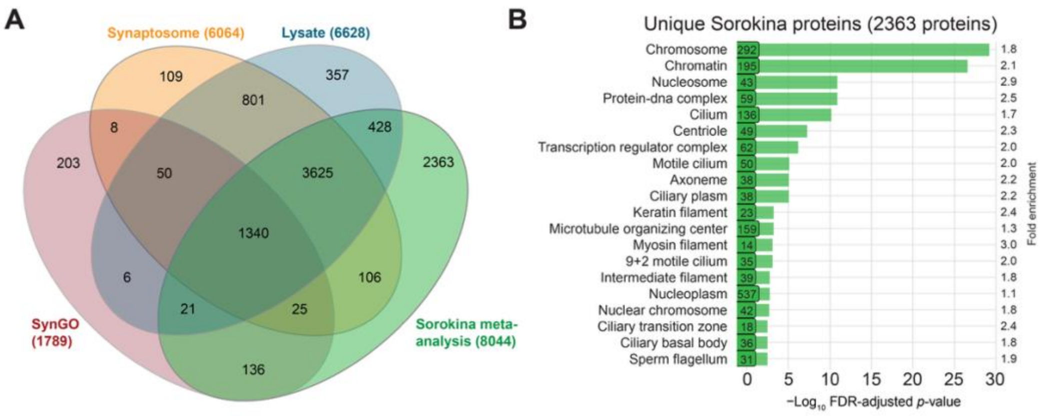

Figure S10. Comparison with a meta-analysis of unique synaptic proteins (based on Sorokina et al. [65]). (A) Venn diagram showing the total number of detected proteins in synaptosome ("Synaptosome"), whole-tissue lysate ("Lysate") samples (cf. Fig 5A) and the 1789 SynGO annotated synaptic proteins ("SynGO") compared with the 8044 unique proteins from the meta-analysis of the synaptic proteome as described by Sorokina et al. [65]. (B) Bar graph showing the top 20 overrepresented CC-GO annotations of the 2363 proteins unique to the meta-analysis of the synaptic proteome using ShinyGO. The number of proteins participating in each annotation, as well as the fold enrichment are indicated. For a complete list of proteins, see Supplemental file S2.

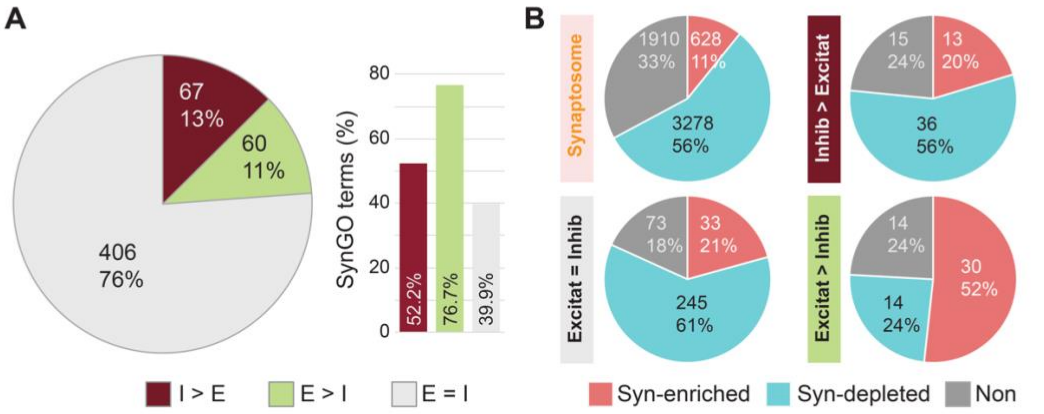

Figure S11. Distribution of proteins related to inhibitory and excitatory cell types (based on Van Oostrum et al. dataset [4]). (A) Pie chart showing distribution of proteins related to inhibitory (I > E), excitatory (E > I) cells and those with no preference (E = I), and bar plot showing their % SynGO v1.3 annotations. (B) Pie charts showing the distribution of synaptosome-enriched, synaptosome-depleted and non-regulated protein terms as detected in the synaptosomes in comparison to that of the markers for the 3 cell types. Numbers and % of total are indicated. For a complete list of proteins in each analysis, see Supplemental file S2.
